# Hugan Qingzhi medication ameliorates free fatty acid-induced L02 hepatocyte endoplasmic reticulum stress by regulating the activation of PKC-δ

**DOI:** 10.1186/s12906-020-03164-3

**Published:** 2020-12-11

**Authors:** Miaoting Yang, Zhijuan Chen, Shijian Xiang, Fan Xia, Waijiao Tang, Xiaorui Yao, Benjie Zhou

**Affiliations:** 1 Department of Pharmacy, People’s Hospital of Longhua, Shenzhen, 518109 Guangdong China; 2 Department of Pharmacy, The Seventh Affiliated Hospital of Sun Yat-sen University, Shenzhen, 518107 Guangdong China; 3 Department of Pharmacy, Zhujiang Hospital, Southern Medical University, Guangzhou, 510282 Guangdong China; 4 Department of Pharmacy, Shantou Central Hospital, Affiliated Shantou Hospital of Sun Yat-sen University, Shantou, 515041 Guangdong China

**Keywords:** Hugan Qingzhi tablets (HQT), Non-alcoholic fatty liver disease (NAFLD), Endoplasmic reticulum stress (ERS), Protein kinase C-δ (PKC-δ)

## Abstract

**Background:**

Previous studies have found that Hugan Qingzhi tablet (HQT) has significant lipid-lowering and antioxidant effects on non-alcoholic fatty liver disease (NAFLD). Moreover, the results of proteomic analysis confirmed that various proteins in endoplasmic reticulum stress (ERS) pathway were activated and recovered by HQT. However, its mechanism remains confused. The purpose of this study was to explore the effects of HQT-medicated serum on hepatic ERS and its relevant mechanisms.

**Methods:**

L02 cells were induced by Free Fatty Acid (FFA) for 24 h to establish a model of hepatic ERS and pretreated with the drug-medicated rat serum for 24 h. Accumulation of intracellular lipid was evaluated using Oil Red O staining and Triglyceride detection kit. The morphological changes of ER were observed by TEM. PKC-δ was silenced by specific siRNA. Western blot and RT-qPCR were applied to detect the expression of markers related to ERS, calcium disorder, steatosis and insulin resistance. The fluorescence of Ca^2+^ influx was recorded using fluorescence spectrophotometer.

**Results:**

HQT-medicated serum significantly decreased the intracellular TG content. Furthermore, it caused significant reduction in the expression of ERS markers and an improvement in ER structure of L02 cells. PKC-δ was activated into phosphorylated PKC-δ in FFA-induced L02 hepatocytes while these changes can be reversed by HQT-medicated serum. Silencing PKC-δ in L02 cells can restore the expression and activity of SERCA2 in ER and down-regulate the expression of IP3R protein to maintain intracellular calcium homeostasis, so as to relieve FFA-induced ERS and its lipid accumulation and insulin resistance.

**Conclusions:**

The results concluded that HQT-medicated serum exerts protective effects against hepatic ERS, steatosis and insulin resistance in FFA-induced L02 hepatocyte. And its potential mechanism might be down-regulating the activation of PKC-δ and stabilization of intracellular calcium.

**Supplementary Information:**

The online version contains supplementary material available at 10.1186/s12906-020-03164-3.

## Background

Nonalcoholic fatty liver disease (NAFLD) has emerged as the worldwide kind of chronic liver disease, which is defined as fatty infiltration of the liver (hepatic steatosis) without viral infection, chronic alcohol intake or any other secondary causes of hepatic injury [1]. Previous exposition of the pathogenesis and progression of NAFLD was the “two-hit” that first put forward by Day et al. As stated by this hypothesis, hepatic steatosis is characterized as the “first hit”, which increases the liver’s susceptibility to various “second hits”, which in turn lead to oxidative stress, inflammation, or cell apoptosis [2, 3].

Recently, mounting evidences have reported that destruction of endoplasmic reticulum (ER) or/and ER stress (ERS) is an important pathway associated with the deterioration of hepatocyte steatosis to Nonalcoholic steatohepatitis (NASH) [4]. Sustained and irreversible ERS plays an essential role in NAFLD pathologic changes which activates multiple signaling pathways [5]. It would interfere with the secretion of triglyceride from liver, indirectly slow down the metabolism of triglycerides by inducing insulin resistance, and then promote the expression of inflammatory factors, and finally deteriorate the progression of NAFLD to NASH [6, 7].

Increasing studies have confirmed that protein kinase C-δ (PKC-δ), one of novel (δ, ε, and θ) PKC isoforms which triggered by diacylglycerol (DAG) acutely and chronically [8], can be involved in the regulation of the course of NASH through ERS pathway. PKC-δ expression was found to be upregulated in human obese and obese diabetic patients to a degree similar to that shown in obese and insulin resistant mice [9, 10]. In a previous study of methionine- and choline- deficient (MCD) dietary induced NASH [11], crucial features for the pathophysiology of NAFLD progression, such as TG accumulation in the liver, oxidative stress and hepatocytes apoptosis, were repressed in PKC-δ^−/−^ mice. Furthermore, fatty acids could activate the expression of PKC-δ accompanied by the activation of ERS [12]. However, an internal link between PKC-δ and the pathology of NASH or ERS remains to be expounded.

Cellular and animal studies have shown that a variety of natural products can prevent or even treat different types of liver diseases [13–16]. Hugan Qingzhi tablet (HQT) has been used in ameliorating NAFLD in clinical practice for a long time. It’s extracted and processed from five plant materials, including *Rh. alismatis*, *Fr. crataegi*, *P. typhae*, *Fo. Nelumbinis*, and *Ra. Notoginseng* [17]. And twelve major compounds in HQT had been certificated and quantified by UHPLC-QqQ-MS spectrometry, namely, alisol A 24-acetate, 23-Oacetylalisol B, typhaneoside, heterosine lisu-3-o-new hesperidin, nuciferine, notoginsenoside R1, isorhamnetin, quercetin, epicatechin, rutin, isoquercetin, and hyperoside [18, 19]. In our previous experiments, it was confirmed that HQT could inhibit intracellular lipid accumulation and oxidative stress by regulating AMPK and PPARα pathway [17, 20]. Furthermore, previous animal experiments have shown that HQT can improve liver lipid metabolism and alleviated hepatocyte inflammation, which involves activating AMPK pathway, inhibiting NF- κB activity and regulating SIRT1 pathway [21–23]. It can prevent the damage caused by long-term high-fat diet in rats and maintain normal protein synthesis and bile metabolism in the liver [24]. Interestingly, proteomics based on relative and absolute quantitative (ITRAQ) suggested that the therapeutic effect of HQT is closely associated with protein processing in the ER, which is obtained not only in the experiment of L02 hepatocyte injury induced by free fatty acids, but also in the NAFLD induced by high-fat diet in rats [25, 26]. However, the mechanisms whereby HQT exerts in ERS remain to be elucidated. In this study we would like to investigate the effects and mechanism of HQT-medicated serum on FFA-induced hepatocyte steatosis model by the activation of ERS.

## Methods

### Plant material and preparation of HQT

HQT (NO: 20170905) was supplied by Guangzhou Chinese Crude Drug Co. (Guangzhou, China) and certified by Chuangming Liu, a professor of Traditional Chinese Medicine in Southern Medical University. Its voucher specimens of HQT have been kept in the Southern Medical University (Guangzhou, China) [17]. The method is divided into following steps: firstly, mixtures of 30% *Rh. alismatis*, 30% *Fr. crataegi*, 15% *P. typhae*, and 20% *Fo. nelumbinis* were extracted by 70% ethanol (6:1, v/w) through heating reflux for 2 h, and then collected the ethanol extracts. The residue was refluxed again according to the aforementioned methods. Then the extracted solution was merged for rotatory evaporation for concentration and dryness. After grinding and sieving, *5% Ra. notoginseng* was added to the dried extracts to prepare HQT. Finally, Thin-layer chromatography (TLC) and High-performance liquid chromatography (HPLC) were applied to analysis the quality and quantity of HQT [18, 19].

### Preparation of HQT-medicated serum

Thirty Sprague Dawley rats (male, weight about 180-220 g) were provided by the Animal Experiment Center of Southern Medical University Guangzhou, China (Quality certificate number: SCXK (Yue) 2011–0015). All animal experiments were authorized by the Southern Medical University Animal Ethics Committee and carried out in compliance with the institutional guidelines (Animal Welfare Assurance L2016133). Those rats were divided into five groups: a vehicle control group, three groups of different doses of HQT-medicated serum (HQT-L, HQT-M, HQT-H) and fenofibrate-medicated serum (FF), and administered orally 1 ml/100 g of saline, different doses of HQT (2.7, 5.4, 10.8 g/kg/day), or fenofibrate (0.4 g/kg/day) respectively. The drug was given intragastrically once a day for a week. After 1 h at the last administrate, animals were injected 2% pentobarbital sodium (3 ml/kg body weight) for anesthesia. And then blood was collected from the abdominal aorta aseptically, centrifuged and stored at − 80 °C. All rats were sacrificed by cervical dislocation under anesthesia. The quality control of HQT- medicated serum have been reported by Yin, etc. [17].

### Experimental protocols

L02 was purchased from China Cell Culture Center (Shanghai, China) and cultured in RPMI-1640 medium containing 10% FBS (Gibco, USA) in a CO_2_ incubator at 37 °C. Cells were treated with FFA (oleic acid and palmitic acid at the ratio of 2:1) at the concentration of 1 mM for 24 h to establish a model of steatosis and ERS. Before cultivation with FFA, different kinds of medicated serum were added into culture medium for 24 h. They were divided into six groups including control group (CON), FFA group (FFA), FFA + 10% low/moderate/high dose of HQT-medicated serum group (HQT-L, HQT-M, HQT-H), and FFA + 10% fenofibrate-medicated serum group (FF) [17].

### Coefficient optimum proportion of HQT-medicated serum

The cytotoxicity of each concentration and medication of the serum on L02 cells was determined and screened by lactate dehydrogenase (LDH) release assay. Cells were cultured with medicated-serum in different concentration (10, 20, 30, 40% of vehicle serum) last for 24 h to find the optimal concentration for therapy. And then cells were added to 10% vehicle serum, 10% low/moderate/high dose of HQT-medication serum, or 10% fenofibrate-medicated serum (selecting the 10% concentration based on previous experiment). After incubating for 24 h, the supernatant was collected and assayed with the LDH colorimetric assay kit.

### Histopathological examination

Cells were washed in PBS, fixed with 4% formaldehyde for 30 min, and stained with solution of Oil Red O for 30 min at room temperature. Images were taken under Olympus BX51 image system (Olympus, Tokyo, Japan) at 400 × magnification.

Cells were fixed with 10% glutaraldehyde for 1 h, washed with PBS for two times and then placed at 4 °C for 4 h. Then cells were dehydrated next in a series concentration of acetone, embedded in epoxy resin and excised into ultrathin sections. The sample sections (CON group, FFA group, HQT-H group) were performed by a transmission electron microscope (FEI Tecnai G^2^20 TWIN, America).

### Biochemical assay

According to the experimental method described as 2.3, the cells were treated with RPMI-1640 containing different concentrations of HQT-medicated serum (HQT-L, HQT-M, and HQT-H) or fenofibrate-medicated serum (FF) last for 24 h, before stimulation with 1 mM FFA for 24 h. Next, the culture supernatant and cells were collected for detecting following biochemical parameters, such as triglycerides (TG), aspartate aminotransferase (AST), alanine aminotransferase (ALT), Reactive oxygen species (ROS) (Nanjing Jiancheng Bioengineering Institute, Nanjing, China), Human IL-1β, Human TNFα (MultiSciences, Hangzhou, China), and Caspase 3 activity (Beyotime Biotechnology, Jiangsu, China). The detected methods were all according to the corresponding manufacturer’s instructions.

### SiRNA transfection of PKC-δ

Cells were plated in 6-well plates without antibiotics. They were divided into six groups including control siRNA group (NC), control siRNA + FFA group (NF), control siRNA + FFA + 10% high dose of HQT-medicated serum group (NH), PKC-δ siRNA group (SC), PKC-δ siRNA + FFA group (SF) and PKC-δ siRNA + FFA + 10% high dose of HQT-medicated serum group (SH). Next, cells with about 50% confluence were transfected with 50 nM PKC-δ siRNA or 50 nM Control siRNA using Lipofectamine 2000 (Life Science, CA, USA). After 6 h, the medium was replaced with RPMI-1640 containing 10% FBS last for 24 h and then subsequent treatments were performed with, or without FFA and HQT-medicated serum. Human PKC-δ siRNA were synthesized and purchased by RiboBio Inc. (Guangzhou, China). The transfected target sequences of PKC-δ siRNA was 5′-GCATGAATGTGCACCATAAdTdT-3′.

### Intracellular calcium levels

According to the experimental method described as 2.3 and 2.7, L02 cells transfected with siRNA and administrated with or without high dose of HQT-medicated serum were washed three times with PBS, incubated with 5 μM of Fluo-4/AM (Beyotime Biotechnology, Jiangsu, China) and Pluronic F-127 (Beyotime Biotechnology, Jiangsu, China) for 30 min at 37 °C and protected from light throughout. The fluorescence of Ca2+ levels in the Fluo-4/AM-treated cells was recorded using Fluorescence spectrophotometer (excitation at 494 nm and emission at 516 nm).

### Capacity of glucose consumption

L02 cells were planted in 96-well at the density of 1 × 10^4^ cells per well. After transfected with siRNA, administrated with medicated serum, and stimulated by FFA, RIMP-1640 containing 100 nmol/L insulin (Procell, Wuhan, China) without phenol red were added into per wells for 6 h and wells without cells acted as Blank group. And then the supernatants were collected and centrifuged at 2500 r/min. Glucose oxidase method (Applygen Technologies Inc., Beijing, China) was used to determine the capacity of glucose consumption.

### Western blot

Cells were lysed by RIPA lysis buffer (KeyGEN BioTECH), centrifuged for 15 min at 12,000 g, and determined by BCA Protein Assay Kit (Beyotime Biotechnology, Jiangsu, China). Proteins were separated by 8–12% SDS/PAGE and transferred to polyvinylidene difluoride membranes. The membrane was blocked with TBS-T (containing 5% bovine serum albumin) for 1 h prior at 25 °C and western blotting with polyclonal antibody at 4 °C overnight. The primary antibodies included rabbit polyclonal antibody against GRP78, PKC-δ, p-PKC-δ, CHOP, CASPASE12 (1:1000, Abcam, UK); SREBP1c, FOXO1, SERCA2, CANX, IP3R, p-PERK, PI3Kp85, AKT, p-AKT, IRE1-α, p-IRE1-α (1:1000, Affinity, USA); PERK, ATF6, ATF4 (1:1000, Cell Signaling Technology, USA); GAPDH (1:5000, Proteintech Group, Inc., USA). After 1 h incubation with corresponding secondary antibody, the immunoreactive bands were visualized by a chemiluminescent substrate (ECL, Amersham, USA).

### RT-qPCR

Trizol reagent (Invitrogen, Carlsbad, USA) was used to extract the total RNA of L02 cells. Quantity and quality of RNA was evaluated by Nanodrop ND-1000 ultraviolet spectrophotometer (Nanodrop). cDNA was reverse-transcribed from 1 μg of total RNA using the Prime Script™ RT Master Mix Reagent kit (TaKaRa). Quantitative real time PCR was carried out using Roche LightCycler480 detection system and SYBR Premix Ex TaqTM kit according to the instruction manual (TaKaRa). Corresponding primers set for PCR are summarized in Table 1.

**Table 1:** Primer design for rtPCR

Gene	Forward	Reverse
GRP78	GTCCTATGTCGCCTTCACTCC	GCACAGACGGGTCATTCCAC
PERK	GGCTTGAAAGCAGTTAG	GGACAGTTGCCTTACAGA
ATF6	GCCGCCGTCCCAGATATTA	GCAAAGAGAGCAGAATCCCA
XBP-1	GAGTTAAGACAGCGCTTGGG	ACTGGGTCCAAGTTGTCCAG
CASPASE-12	ACAGCACATTCCTGGTGTTTATG	CAGACTCTGGCAGTTACGGTTG
CHOP	GGAAACAGAGTGGTCATTCCC	CTGCTTGAGCCGTTCATTCTC
PKC-δ	GAAGCAGGGATTAAAGTGTG	TTCTTCTCGAAACCCTGATA
β-ACTIN	TGACGTGGACATCCGCAAAG	CTGGAAGGTGGACAGCGAGG

### Statistical analysis

All data presented as mean ± standard deviation. Statistical significance differences between groups were evaluated by using Student’s t-test or One-way analysis of variance (ANOVA) with the SPSS 23.0 statistical software package. When *P* value is less than 0.05, the difference is considered to be statistically significant.

## Results

### HQT-medicated serum reduces FFA-induced L02 hepatocyte steatosis

**Fig. 1:**
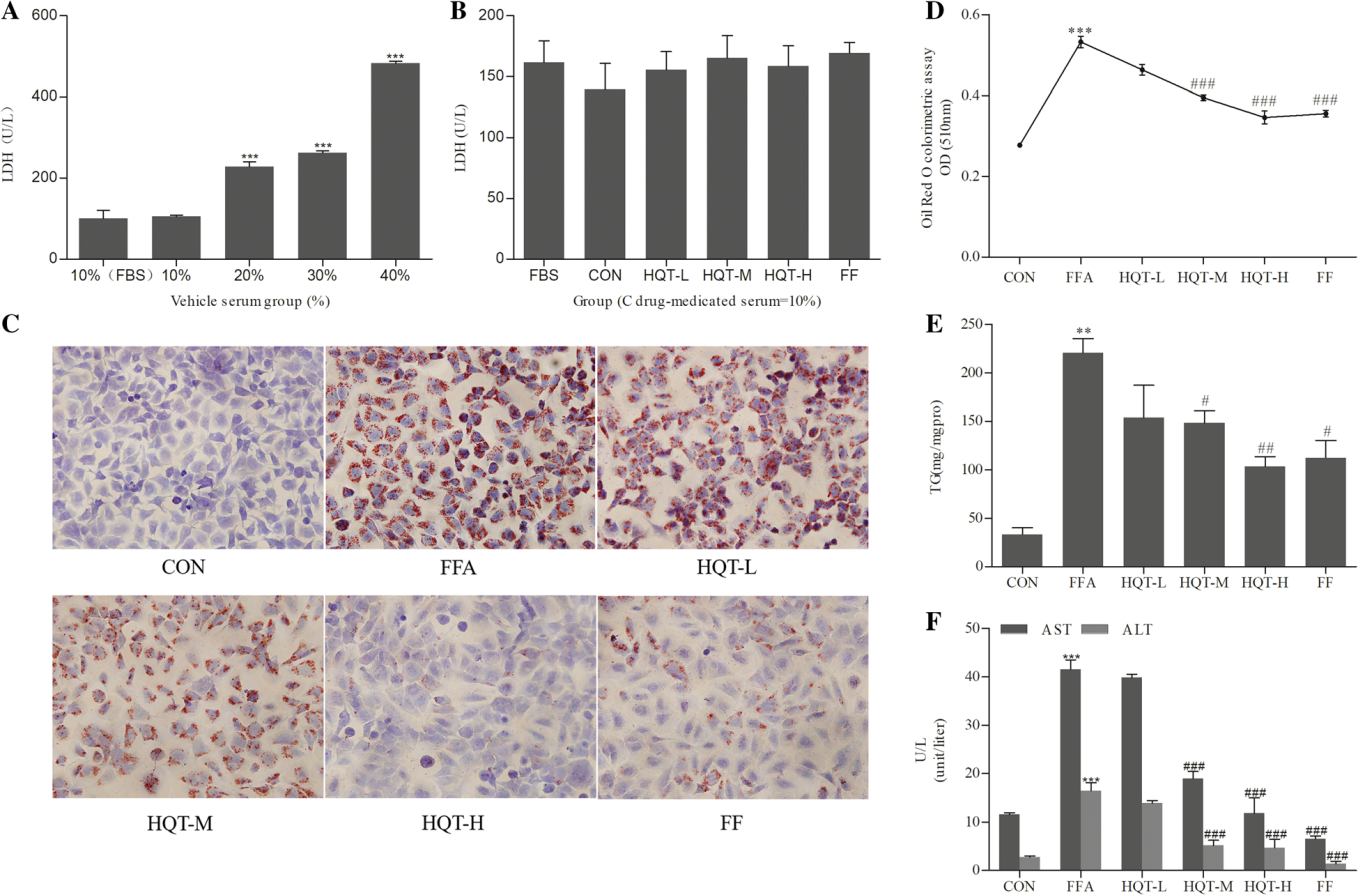
HQT-medicated serum reduces FFA-induced L02 hepatocyte steatosis. **a** and **b** LDH release assay for each medicated serum treatment in FFA-induced L02 cells. L02 cells were treated with or without different concentrations of blank vehicle serum (0,10,20,30,40%) for 24 h (**a**); L02 cells were treated with HQT-L, HQT-M, HQT-H, FF and vehicle serum, respectively (**b**). **c** Lipid droplets were observed by Oil Red O staining (original magnification: × 400). **d** Lipid content in the cells determined by Oil red-based colorimetric assay. **e** TG content in FFA-induced L02 hepatocytes. **f** AST and ALT levels in FFA-induced L02 hepatocytes. Results are expressed as means ± S.D. ^***^p < 0.001 compared with the 10% FBS group. ^**^p < 0.01, ^***^p < 0.001 vs CON group, ^#^*p* < 0.05, ^##^*p* < 0.01, ^###^*p* < 0.001 vs FFA group

In order to examine the optimum concentration of HQT serum for L02 hepatocyte, we performed a LDH release assay to measure the cytotoxicity of HQT-medicated rat sera. In comparison to normal culture medium containing 10% FBS, 10% vehicle serum have no harm to L02 cells (*p* > 0.05, Fig. 1a), however, when the concentration reached over 20% the content of LDH have increased sharply (*p* < 0.001, Fig. 1a). Furthermore, the results also showed that the treatments of 10% each dose of HQT-medicated serum and 10% fenofibrate-medicated serum have no significant difference on LDH release (*p* > 0.05, Fig. 1b), signifying that each medicated serum administration at the concentration of 10% has no toxicity to L02 hepatocytes.

After intervention with 1 mM FFA for 24 h, HQT-medicated serum showed the similar ability as fenofibrate serum group to attenuate FFA-induced intracellular fatty degeneration in L02 hepatocytes. The size and number of intracellular lipid droplets increased remarkably in the FFA group (Fig. 1c) whereas treatment with HQT-medicated serum have less intracellular fatty lesions which committed with the data of oil red-based colorimetric assay (Fig. 1d). Besides, the content of TG in FFA group was much higher than that in CON group (*p* < 0.01, Fig. 1e), and reduced significantly in HQT-M, HQT-H, and FF group (*p* < 0.05, 0.01, 0.05, Fig. 1e). Level of AST and ALT in the supernatant of culture medium are used to evaluate the functional status of L02 cells. The results showed that AST and ALT levels of HQT-M group, HQT-H group and FF group were significantly lower than those in FFA group (*p* < 0.001, Fig. 1f).

### HQT-medicated serum protects against FFA-induced L02 hepatocyte from ERS

**Fig. 2:**
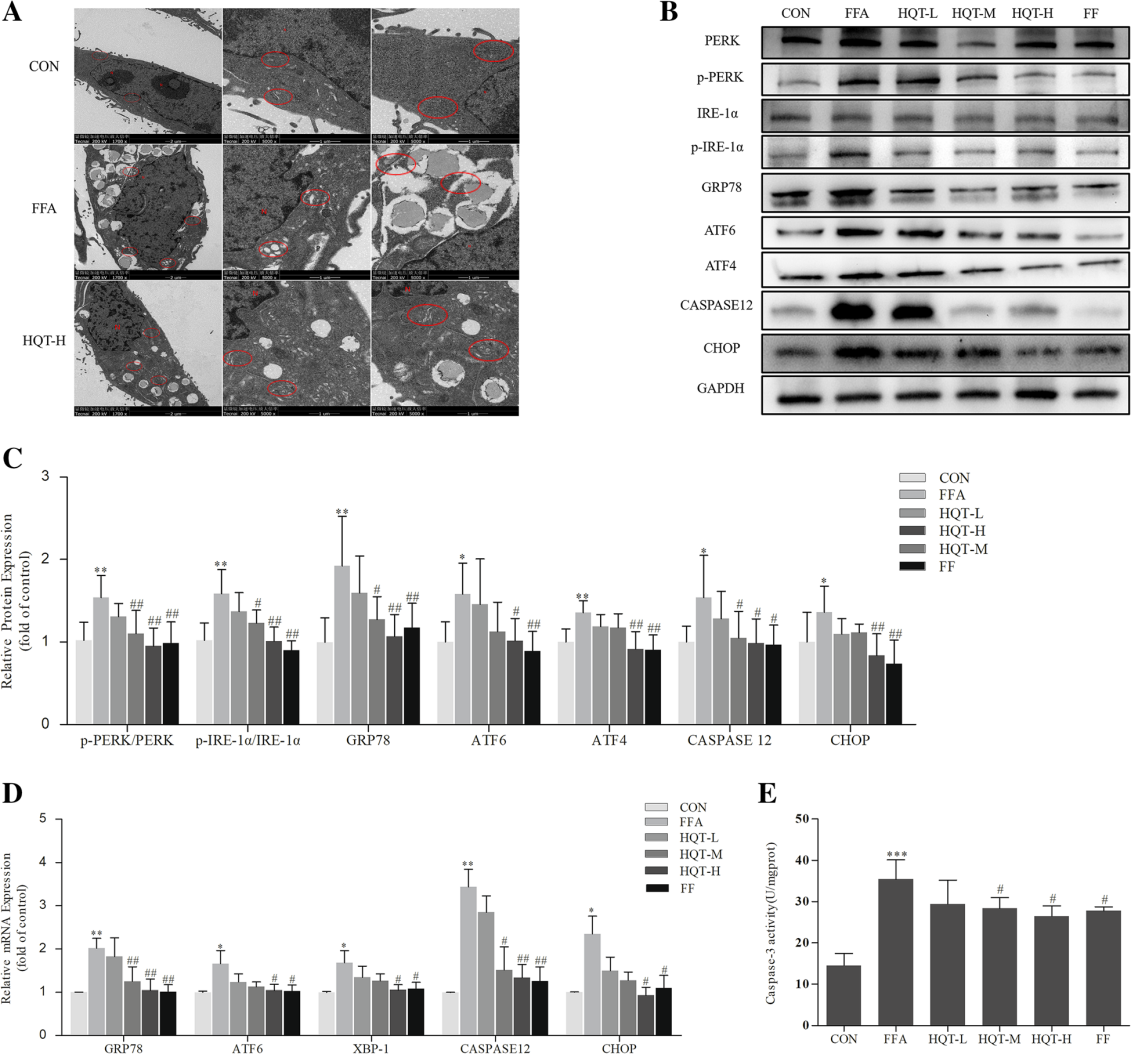
HQT-medicated serum protects against FFA-induced L02 hepatocyte from ERS. **a** Transmission electron microscopy of L02 hepatocytes from control group showing normal Endoplasmic reticulum (er) architecture, from FFA group and HQT-H group showing ER dilatation and degranulation (d), lipid vacuoles (w), and inflammatory (star) (× 1700, × 5000). **b**, **c** and **d** The expression levels of PERK, p-PERK, IRE-1α, p-IRE-1α, GRP78, ATF6, ATF4, CASPASE12, CHOP protein and the expression levels of GRP78, ATF6, XBP-1, CASPASE12, CHOP mRNA in FFA-stimulated L02 hepatocytes. **e** Caspase-3 activity in FFA-induced L02 hepatocytes. Results are expressed as mean ± S.D. ^*^p < 0.05, ^**^p < 0.01, ^***^p < 0.001 vs CON group, ^#^*p* < 0.05, ^##^*p* < 0.01, ^###^*p* < 0.01 vs FFA group

To evaluate the effects of HQT-medicated serum on FFA-induced L02 hepatocyte ERS, the substructure of endoplasmic reticulum was firstly performed by transmission electron microscopy. Endoplasmic reticulum was arranged in an orderly and normally manner with ribosomes attaching to its surface neatly in CON group, and there were no obvious lipid vesicles found in the cytoplasm. But the substructure of ER in FFA group showed severe damage as evidenced by rough ER dilatation and degranulation. And lipid droplets of different sizes were distributed in the cytoplasm, which led to the disorder of the distribution of the ER and structural damage. However, after the intervention of high dose of HQT-medicated serum, the overall structure of the cells tended to be normal that the phenomenon of lipid vacuoles was significantly reduced and the structure and morphology of the ER were significantly improved (Fig. 2a).

Secondly, the function of ER in different groups were performed in Western blot and RT-qPCR analysis by detecting the expression of ERS-related proteins and mRNA. The expression levels of p-PERK, p-IRE-1, GRP78, ATF6, ATF4, CHOP, and CASPASE12 protein was upregulated in FFA-stimulated L02 cells compared by CON group (*p* < 0.05, Fig. 2b and c). However, L02 cells pretreated with HQT-M-, HQT-H- or fenofibrate-medicated serum all showed significantly lowered expression levels of ERS-related proteins compared by FFA group (*p* < 0.05, Fig. 2b and c). Besides, corresponding RT-qPCR results presented in Fig. 2d were consistent with the western blot validation.

Cysteine-requiring Aspartate Protease (Caspase) is a family of proteases in the process of cell apoptosis and Caspase-3 is the last executor of the Caspase apoptosis pathway induced by ERS, therefore the activity of Caspase-3 enzyme was next detected for identifying the protective effects of HQT on the pathway of ERS-induced apoptosis. The activity of Caspase-3 in FFA group was 2.4 times higher than that in CON group (*p* < 0.001, Fig. 2e). However, pretreatment of the cells with HQT-M, HQT-H or FF have reduced these enzyme activity (*p* < 0.05).

### PKC-δ silencing ameliorates FFA-induced L02 hepatocyte ERS

**Fig. 3:**
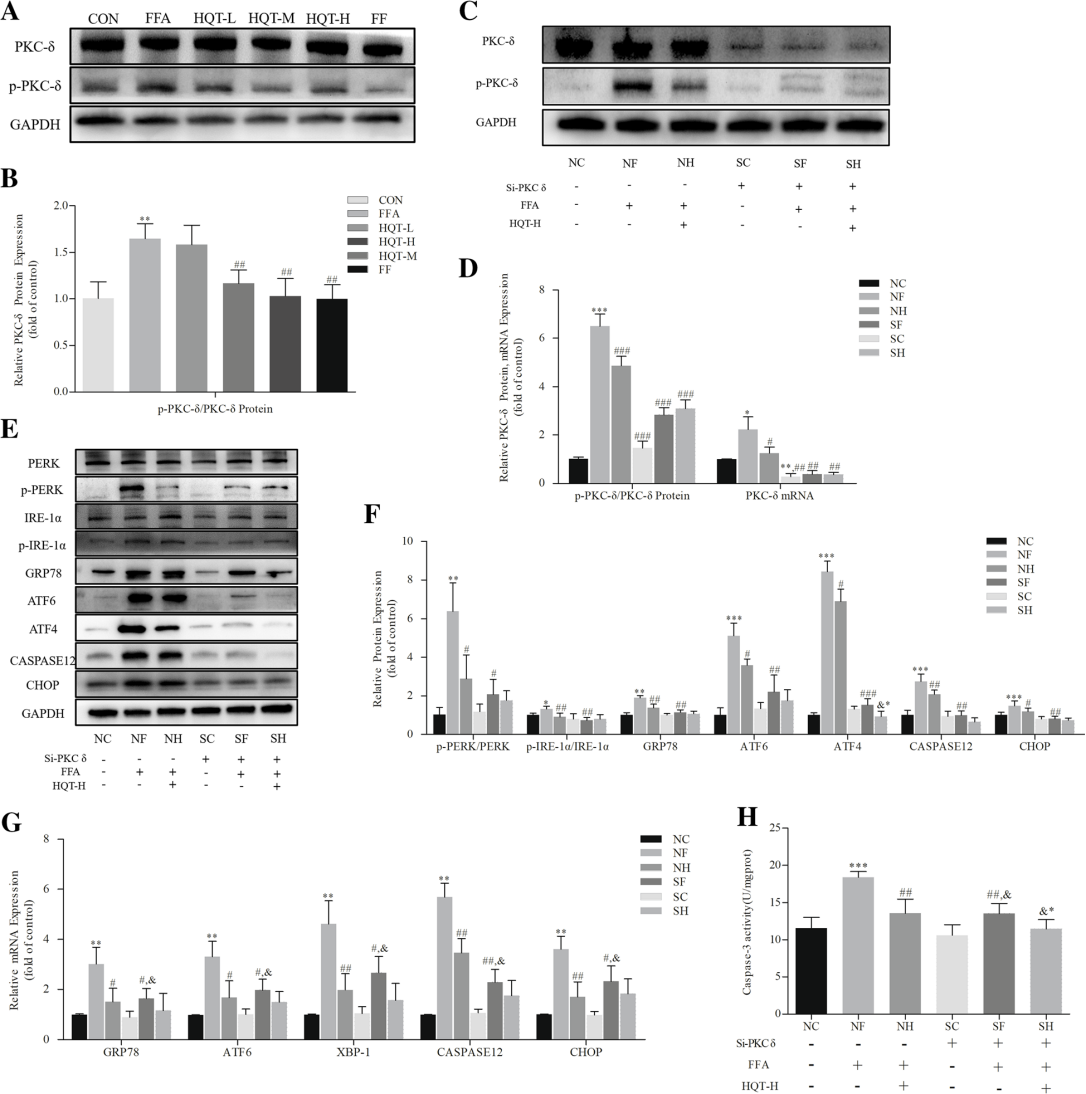
PKC-δ silencing ameliorates FFA-induced L02 hepatocyte ERS. control siRNA group (NC), control siRNA + FFA group (NF), control siRNA + FFA + 10% high dose of HQT-medicated serum group (NH), PKC-δ siRNA group (SC), PKC-δ siRNA + FFA group (SF) and PKC-δ siRNA + FFA + 10% high dose of HQT-medicated serum group (SH). **a** and **b** Effects of HQT-medicated serum on the expression levels of total and phosphorylated PKC-δ protein and its mRNA in FFA-stimulated L02 cells. **c** and **d** Expression of total and phosphorylated PKC-δ protein and its mRNA after treatment with PKC-δ siRNA in different group of L02 cells. **e**, **f** and **g** Effects of HQT-medicated serum on the expression levels of p-PERK, p-IRE-1α, GRP78, ATF6, ATF4, CASPASE12, CHOP protein and the expression levels of GRP78, ATF6, XBP-1, CASPASE12, CHOP mRNA in FFA-stimulated L02 hepatocytes with or without transfection of PKC-δ siRNA. **h** Activity of Caspase-3 in FFA-stimulated L02 hepatocytes with or without transfection of PKC-δ siRNA. Results are expressed as mean ± S.D. ^*^p < 0.05, ^**^p < 0.01 vs CON group, ^#^*p* < 0.05, ^##^*p* < 0.01 vs FFA group. ^*^p < 0.05, ^**^p < 0.01, ^***^p < 0.001 vs NC group; ^#^*p* < 0.05, ^##^*p* < 0.01, ^###^*p* < 0.001 vs NF group; ^&^*p* < 0.05, ^&&^*p* < 0.01, ^&&&^*p* < 0.001, vs SC group; ^&*^ *p* < 0.05, ^&&*^ *p* < 0.01, ^&&*^ *p* < 0.01 vs SF group

In order to figure out the mechanism of HQT-medicated serum in protecting against FFA-induced L02 hepatocyte from ERS, surprisingly, we found that the phosphorylation of PKC-δ increased notably in the FFA group compared by CON group (*p* < 0.01, Fig. 3a and b), but was downregulated in HQT-M, HQT-H and FF group (*p* < 0.01, 0.01, 0.01).

Whether HQT can alleviate FFA-induced ERS by downregulating the phosphorylated expression of PKC-δ? Therefore, PKC-δ siRNA was applied to L02 cells for exploring the impact of PKC-δ inhibition in FFA-stimulated ERS in L02 hepatocytes. The protein and mRNA expression levels of PKC-δ was significantly decreased in L02 cells after PKC-δ siRNA treatment. And the phosphorylated expression of PKC-δ was downregulated at the same time (*p* < 0.001, Fig. 3c and d). FFA treatment in the control siRNA group upregulated the expression of p-PERK, p-IRE-1α, GRP78, ATF6, ATF4, CHOP, and CASPASE12 protein (*p* < 0.01, Fig. 3e and f). However, transfection of PKC-δ siRNA protected FFA-induced L02 cells from ERS by downregulating ERS-related proteins or mRNA expression. Additionally, our results shown in Fig. 3h indicated that PKC-δ silencing inhibited Caspase-3 activity of L02 cells induced by FFA. FFA treatment led to activating the enzyme of Caspase-3 (*p* < 0.001). However, the inhibition of PKC-δ by siRNA significantly decreased the influence of FFA on Caspase-3 activity (*p* < 0.01).

### PKC-δ silencing recovers calcium homeostasis in ER

**Fig. 4:**
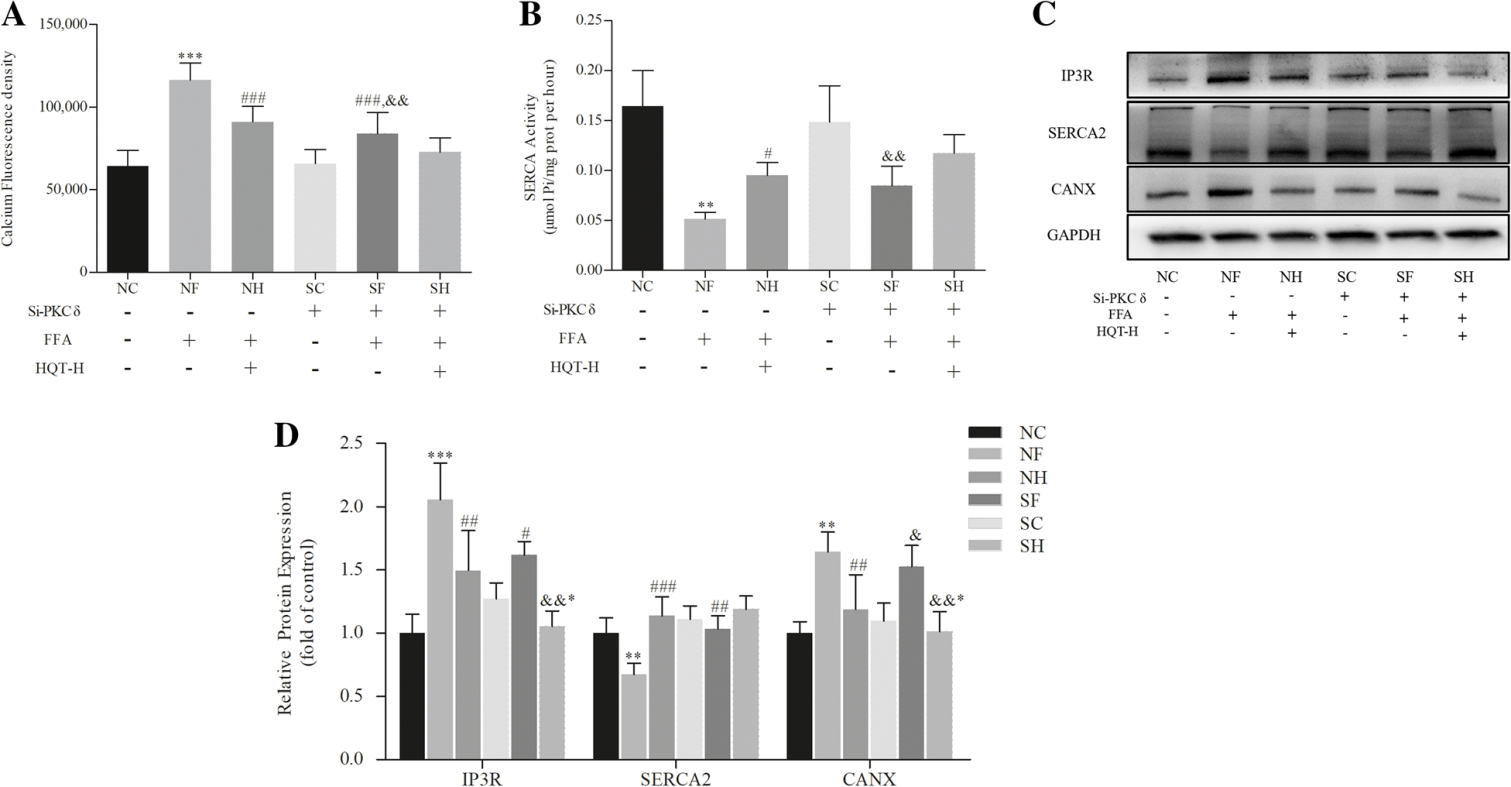
PKC-δ silencing recovers Calcium homeostasis in ER. control siRNA group (NC), control siRNA + FFA group (NF), control siRNA + FFA + 10% high dose of HQT-medicated serum group (NH), PKC-δ siRNA group (SC), PKC-δ siRNA + FFA group (SF) and PKC-δ siRNA + FFA + 10% high dose of HQT-medicated serum group (SH). **a** The changes of calcium fluorescence densities were monitored in Fluo-4/AM-loaded L02 cells. **b** Effects of HQT-medicated serum on SERCA activity induced by FFA in normal L02 cells and PKC-δ knockdown L02 cells. **c** and **d** The expression levels of SERCA2, CANX, and IP3R protein in L02 cells with or without transfection of PKC-δ siRNA. Results are expressed as mean ± S.D. ^*^p < 0.05, ^**^p < 0.01, ^***^p < 0.001 vs NC group; ^#^*p* < 0.05, ^##^*p* < 0.01, ^###^*p* < 0.001 vs NF group; ^&^*p* < 0.05, ^&&^*p* < 0.01, ^&&&^*p* < 0.001, vs SC group; ^&*^ *p* < 0.05, ^&&*^ *p* < 0.01, ^&&*^ *p* < 0.01 vs SF group

Calcium imbalance in endoplasmic reticulum is decisive for the progress of ER Stress. Fluo-4/AM, a calconcarboxylic acid dye, was used to determine differences in the calcium concentration in L02 cells with different treatments. FFA treatment increased the level of cellular calcium (*p* < 0.001, Fig. 4a), but these changes were attenuated moderately in PKC-δ knockdown L02 cells (*p* < 0.001). And there is no significant difference between SF group and SH group (*p* > 0.05). Otherwise, SERCA is one of the essential enzymes in keeping balance of ER calcium. In comparison of the NC group, SERCA activity was obviously repressed in the NF group with FAA treatment (*p* < 0.01, Fig. 4b). And PKC-δ downregulation partially restored the SERCA activity induced by FFA, but there was no difference between SF group and NF group (*p* > 0.05). Additionally, in comparison with the NC group, the protein of CANX and IP3R that associated with calcium transport were upregulated by FFA treatment alone, whereas SERCA2 was downregulated (*p* < 0.01, Fig. 4c and d). And the expression levels of IP3R and SERCA2 protein were reversed effectively (*p* < 0.01), but the expression of CANX showed no significant difference after transfected with PKC-δ siRNA (*p* > 0.05).

### PKC-δ silencing inhibits FFA-induced L02 hepatocyte steatosis

**Fig. 5:**
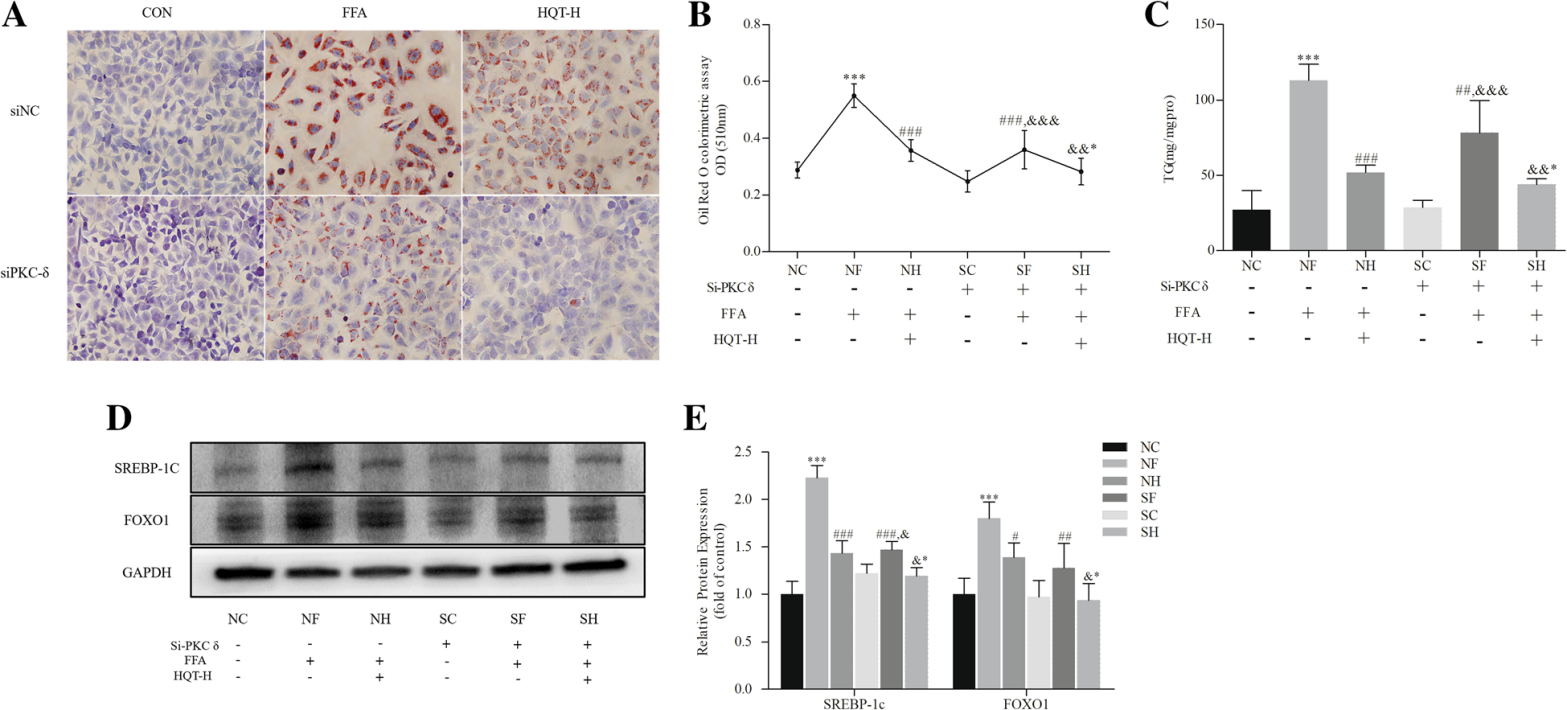
PKC-δ silencing inhibits FFA-induced L02 hepatocyte steatosis. Control siRNA group (NC), control siRNA + FFA group (NF), control siRNA + FFA + 10% high dose of HQT-medicated serum group (NH), PKC-δ siRNA group (SC), PKC-δ siRNA + FFA group (SF) and PKC-δ siRNA + FFA + 10% high dose of HQT-medicated serum group (SH). **a** Oil Red O staining (original magnification: × 400). **b** Lipid content in the cells determined by Oil red-based colorimetric assay. **c** TG (triglyceride) content. **d** and **e** The expression levels of SREBP-1C and FOXO1 protein in L02 cells with or without transfection of PKC-δ siRNA. Results are expressed as mean ± S.D. ^*^p < 0.05, ^**^p < 0.01, ^***^p < 0.001 vs NC group; ^#^*p* < 0.05, ^##^*p* < 0.01, ^###^*p* < 0.001 vs NF group; ^&^*p* < 0.05, ^&&^*p* < 0.01, ^&&&^*p* < 0.001, vs SC group; ^&*^ *p* < 0.05, ^&&*^ *p* < 0.01, ^&&*^ *p* < 0.01 vs SF group

L02 cells with PKC-δ gene knockdown showed the ability to inhibit intracellular lipid accumulation induced by FFA. Hepatocyte steatosis induced by FFA pretreated with control siRNA could still lead to a substantial increase in TG production, whereas pretreatment of PKC-δ siRNA significantly relieved these symptoms (Fig. 5a, b and c). Another finding that is consistent with those presented in Fig. 5d and e, FFA treatment resulted in the upregulation of SREBP-1C and FOXO1. But that were significantly downregulated in PKC-δ knocked down cells. Furthermore, in comparison of SF group, L02 hepatocyte transfected with PKC-δ siRNA and pretreated of HQT-medicated serum demonstrating a better efficacy in FFA-induced L02 hepatocyte steatosis (*p* < 0.05, Fig. 5b, c, d, and e).

### PKC-δ silencing improves insulin resistance in FFA-induced L02 hepatocyte

**Fig. 6:**
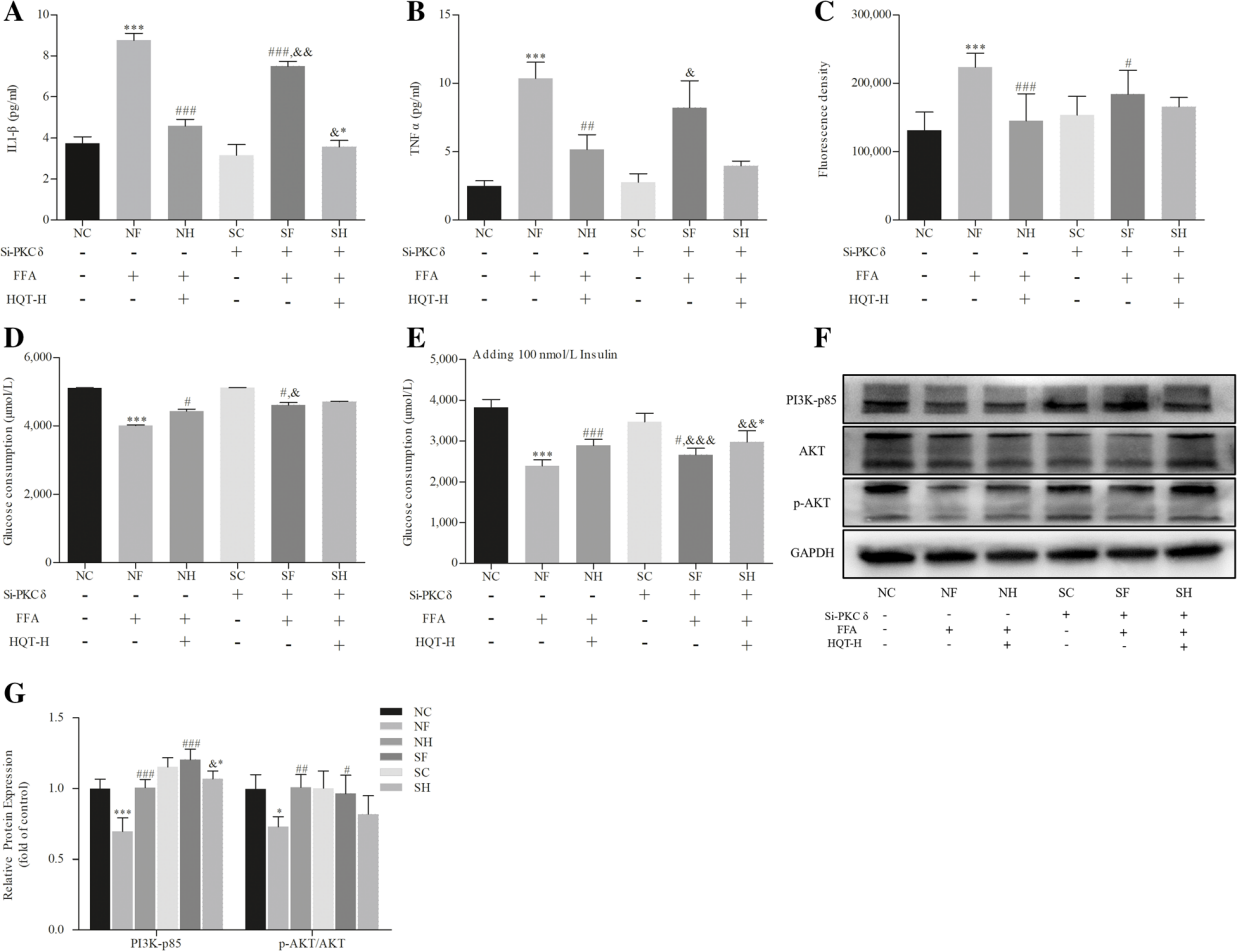
PKC-δ silencing improves insulin resistance in FFA-induced L02 hepatocyte. Control siRNA group (NC), control siRNA + FFA group (NF), control siRNA + FFA + 10% high dose of HQT-medicated serum group (NH), PKC-δ siRNA group (SC), PKC-δ siRNA + FFA group (SF) and PKC-δ siRNA + FFA + 10% high dose of HQT-medicated serum group (SH). **a** and **b** Levels of IL-1β and TNFα induced by FFA in L02 cells with or without transfection of PKC-δ siRNA. **c** ROS level. **d** and **e** Glucose consumption with/without 100 nmol/L insulin treatments. **f** and **g** The expression levels of PI3K-p85, AKT, and p-AKT protein in L02 cells with or without transfection of PKC-δ siRNA. Results are expressed as mean ± S.D. ^*^p < 0.05, ^**^p < 0.01, ^***^p < 0.001 vs NC group; ^#^*p* < 0.05, ^##^*p* < 0.01, ^###^*p* < 0.001 vs NF group; ^&^*p* < 0.05, ^&&^*p* < 0.01, ^&&&^*p* < 0.001, vs SC group; ^&*^ *p* < 0.05, ^&&*^ *p* < 0.01, ^&&*^ *p* < 0.01 vs SF group

Levels of IL-1β, TNF-α, ROS and glucose consumption were performed to evaluate the severity of insulin resistance in FFA-induced L02 hepatocyte. PKC-δ silencing have attenuated FFA-induced IL-1β and ROS production (*p* < 0.001, 0.05, Fig. 6a and c). Nevertheless, TNF-α showed no significant different between FFA-induced L02 cells with the pretreatments of PKC-δ siRNA and control siRNA (*p* > 0.05, Fig. 6b). Additionally, in PKC-δ knocked down cells, the attenuate capacity of glucose consumption induced by FFA were recovered significantly (*p* < 0.05, Fig. 6d and e). Lastly, we evaluated the expression levels of PI3K-p85, AKT, and p-AKT that associated with cellular insulin utilization. Results showed that the protein of PI3K-p85 and p-AKT/AKT were decreased in FFA-induced L02 cells without PKC-δ siRNA transfection(*p* < 0.001,0.05, Fig. 6f and g). However, PKC-δ silencing by siRNA significantly ameliorated the effects of FFA on the expression of PI3K-p85 and p-AKT/AKT. (*p* < 0.05, 0.05, Fig. 6f and g).

## Discussion

NAFLD is a reversible disease in its early stage. Therefore, treatments should be carried out as soon as possible to prevent it from turning into NASH, a more aggressive condition and inflection point of NAFLD, and to obtain better prognosis [27]. Accumulating studies have found that abnormal expression of ERS-related proteins, such as GPR78, p-PERK, and ATF6, were detected in liver cells of patients with obesity or NAFLD [28], which were involved in the pathology process of NAFLD, including hepatic steatosis, insulin resistance, inflammation and apoptosis.

Proteomic analysis had showed that the antagonistic effect of HQT on FFA-induced injury of L02 hepatocytes was related to the protein processing of ER [25]. However, it is still not fully clarified. In this study, we prepared HQT-medicated serum according to the theory of serum pharmacology [29]. Then, we came to the same conclusion as before that the 10% (vol/vol) HQT-medicated sera has no toxicity in culturing L02 cells through an LDH-released assay [17]. Next, to establish a cell model of lipid-overload and ER Stress, the L02 cells were exposed to FFA at a final concentration of 1 mM for 24 h determined by results from oil red O staining, TG content, expression levels of ERS-related protein or mRNA (Additional file 1). High and moderate concentrations of HQT serum showed the similar ability as fenofibrate serum group to attenuate FFA-induced intracellular TG and lipid droplets. In addition, AST and ALT activities were decreased in HQT groups indicating its beneficia effect on hepatoprotective effect.

Excessive lipids accumulated in the hepatocytes results in hepatic steatosis, then the generation of ROS and inflammatory cytokines would be stimulated, which cooperate to promote NAFLD progressing to NASH [30, 31, 32]. Along with that process, the disturbances in ER plays an essential role in NASH development, exacerbation of hepatic steatosis, IR, and inflammatory response by inducing hepatocyte apoptosis [33, 34]. Our results showed that upregulation of signaling molecules such as GRP78, p-PERK, p-IRE-1α, ATF6 and ATF4 in the FFA-treated group suggested that ERS model in vitro was successfully induced by FFA. There are a variety of hazards inducing ERS in the development process of NAFLD, such as lipid-overload, hypercholesterolemia, oxidative stress and inflammatory [35], etc. Simultaneously, multiple pathways of ERS promotes the transformation of NAFLD to NASH. The pathways of PERK/ATF4, IRE1/XBP1, ATF6 contribute to the expression of adipose genes to regulate the synthesis, differentiation and transportation of lipid. PERK deletion inhibited the sustained expression of FAS, ACL and SCD1 in immortalized murine embryonic fibroblasts [34, 36]. ATF4 overexpression induces early onset of hyperlipidemia in zebrafish [36], while hepatic lipogenesis was diminished in fructose-fed ATF4-deficient mice with impacts on downregulation of PPAR-γ, SREBP-1, ACC and FAS [37]. Activated XBP1 and ATF6 can directly combined with fat synthesis gene promoter for regulating the assembly and secretion of VLDL [38–41]. Additionally, activated IRE-1α and PERK mediates inflammatory reaction through the pathways of JNK/AP-1 and IKK/NF-κΒ to upregulate a variety of inflammatory factors including TNF-α、IL-1β and MCP-1, which were subsequent to hepatic IR, inflammation, and even organic damage [42]. Similarly, HQT serum or fenofibrate serum all showed significantly lowered protein or mRNA expression levels of ERS-related signaling molecular which can be concluded that HQT serum or fenofibrate serum protected hepatocytes against FFA-induced ERS, thus effectively improving its pathologic and functional state. Moreover, under a long-term period of ERS, cell apoptosis can be mediated by signaling pathway of PERK/ATF4/CHOP, IRE-1/JNK and CASPASE12 [43–45]. On the one hand, antiapoptotic proteins are reduced, while proapoptotic proteins are increased [46]. On the other hand, activation of calcium efflux receptor causes calcium leakage in the endoplasmic reticulum, then increases calcium concentration in the cytoplasm, and generation of oxygen free radicals, which ultimately activates cell apoptosis by the mitochondrial dependent or independent pathways [47–49]. Surprisingly, our results proved that ERS-induced apoptosis has occurred in FFA group with upregulated expression of CHOP and CASPASE12, while HQT serum or fenofibrate serum not only lowered protein or mRNA expression levels of them, but also protected hepatic endoplasmic reticulum against FFA-caused vesicular dilation, ribosome shedding, and other abnormal structural changes, and reduced the generation of lipid vacuoles, so that the overall structure of L02 cells tend to be normal.

Mounting researches has focused on the role of PKC-δ, an atypical isoform of the PKC family, which are signal transduction enzymes activated by diacylglycerol (DAG), a metabolite of free fatty acid [50]. Previous studies have confirmed that PKC-δ can be involved in the regulation of the course of NASH through ERS pathway, and downregulated expression of PKC-δ have been useful in reducing the expression levels of ERS-related molecules, decreasing concentration of blood lipid and ALT in NASH or diabetic mice, in order to improving hepatic steatosis and fibrosis [12, 51–53]. Notably, we observed the activation of phosphorylated PKC-δ in FFA-induced L02 hepatocytes while these changes can be reversed by HQT-medicated serum. And whether HQT can alleviate FFA-induced ERS by regulating the activation of PKC-δ? Therefore, we used specific siRNA sequences to silence PKC-δ gene in L02 cells. Our results showed that the expression levels of p-PERK, p-IRE-1α, GRP78, ATF6, CASPASE12 and CHOP protein in SF group were not significantly different from those in SH group except ATF4 after treated with FFA for 24 h, however, the cells in the PKC-δ siRNA-transfected group were significantly lower than those in the non-transfected group. In addition, PKC-δ silencing could effectively inhibit the activation of Caspase-3 in L02 cells induced by FFA, but the Caspase-3 activity in the SF group was still increased with a comparison to the SC group. The results indicated that PKC-δ silencing can significantly ameliorate FFA-induced ERS in L02 hepatocytes.

**Fig. 7:**
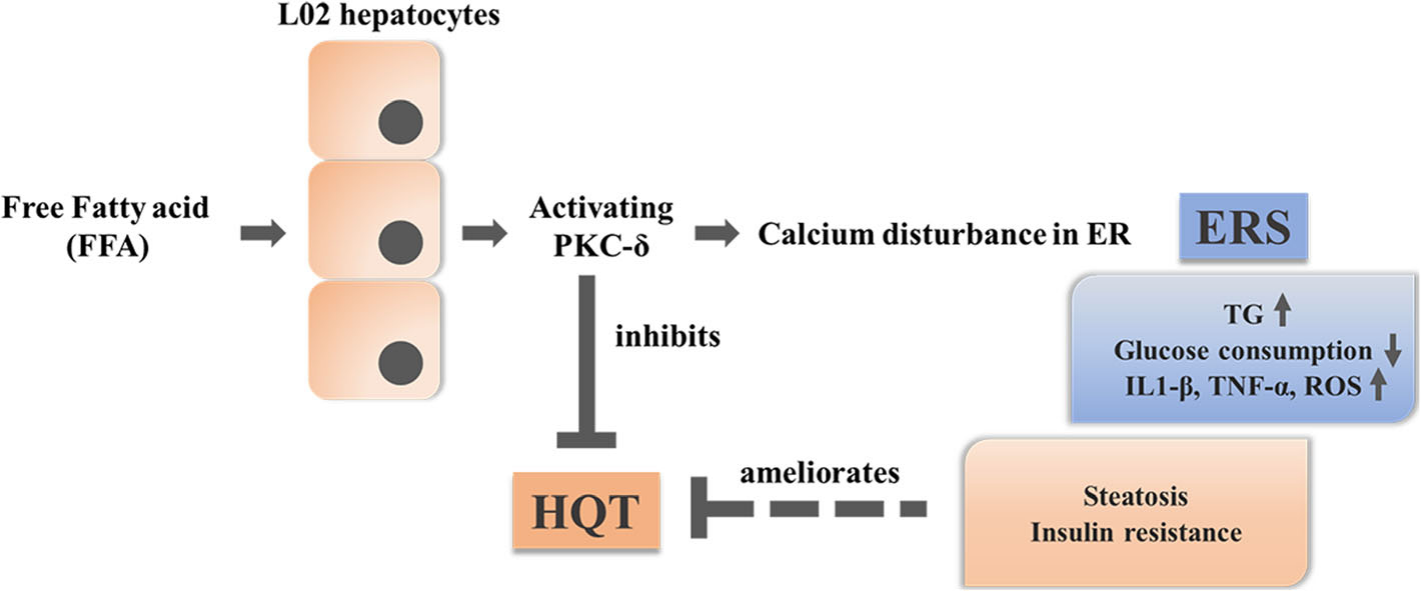
HQT-medicated serum ameliorates Free Fatty Acid-Induced L02 hepatocyte endoplasmic reticulum stress by regulating the activation of PKC-δ. HQT-medicated serum can downregulate the expression of ERS-related signaling molecules, so that effectively protected against hepatocytes from the FFA-induced steatosis and improved its functional status. HQT-medicated serum can inhibit the activating of PKC-δ in L02 cells to maintain intracellular calcium homeostasis, thus relieving the FFA-induced ERS and its lipid accumulation and insulin resistance. ↑: up-regulation; ↓: down-regulation

And how does PKC-δ relate to ERS? Endoplasmic reticulum, as the main site of calcium storage, maintains intracellular calcium homeostasis. Under physiological conditions, calcium ions are transferred to the cytosol through the Ryanodine Receptor (RyR) and 1,4,5-trisphosphate receptor (IP3R) [54], and transported into the ER through SERCA to maintain the dynamic balance of Ca^2+^ in the ER. Inhibition of SERCA activity leads to an increase in Ca^2+^ concentration in cytoplasm, which in turn activates ERS, CaMKKβ/AMPK/mTOR cascade signaling pathways, and ultimately promotes cell apoptosis [55, 56]. In addition, the inhibition of SERCA resulted in the production of ERS and IR in ob/ob mice fed with a western diet [57]. Conversely, enhancing the activity of SERCA blocked the ERS-activated apoptotic pathway induced by palmitic acid in BEL-7402 hepatoma cell line [58]. These results indicated that SERCA inactivation is closely related to the production of ERS and plays an important role in metabolic disorder through ERS. In present experiment, it showed that the activity of SRECA2 decreased significantly, and intracellular calcium concentration increased significantly in L02 cells treated with FFA, while the expression level and activity of SERCA2 in L02 cells transfected with PKC-δ siRNA were restored. Meanwhile, the expression of IP3R and CANX decreased obviously to maintain intracellular calcium homeostasis. It can be seen that silencing PKC-δ may play a key role in maintaining calcium homeostasis. The pathogenic factors of NAFLD are closely related to IR. Excessive accumulation of lipid in hepatocytes can disrupt the transduction of insulin signaling pathway, and IR can also affect the synthesis, secretion and transport of lipid in the liver. Previous studies have found that the expression of SERCA in hepatocytes and macrophages of obese mice is decreased with IR, and the expression of ERS signaling molecules is upregulated, but recovery of SERCA2 can alleviate ERS, normalize content of blood glucose and hepatic TG, and even facilitate transduction of insulin [59].In addition, activity of SERCA2 protein of islet β cells in diabetic patients were decreased through NO−/AMPK-dependent pathways under inflammatory response, which could result in glucose intolerance and reduce islet β cell proliferation and insulin secretion. Then, ERS was induced at the same time [60]. Similarly, silencing PKC-δ in L02 cells restored the expression and activity of SERCA2 protein and alleviated status of ERS. After remission of ERS, on the one hand, the expression levels of SREBP-1c and FOXO-1 protein were down-regulated for alleviating FFA-induced steatosis; On the other hand, silencing PKC-δ inhibited the production of inflammatory factors (IL-1β and TNF-α) and ROS and up-regulated the expression of PI3K-p85 and p-AKT protein in order to restoring the transduction pathway of insulin, thus alleviating IR and glucose intolerance in FFA-induced L02 hepatocytes (Fig. 7).

## Conclusions

HQT-medicated serum could effectively alleviate the ERS state of L02 cells. On the one hand, it significantly decreased the intracellular TG content and the activities of AST and ALT. HQT-medicated serum can downregulate the expression of ERS-related signaling molecules, so that effectively protected against hepatocytes from the FFA-induced steatosis and improved its functional status. Moreover, the mechanism may be related to regulating the activation of PKC-δ. PKC-δ plays an important role in the progression of NAFLD disease. Silencing PKC-δ in L02 cells can restore the expression and activity of SERCA2 protein in ER and downregulate the expression of IP3R protein to maintain intracellular calcium homeostasis, thus relieving the FFA-induced ERS and its lipid accumulation and insulin resistance.

## Supplementary Information

**Additional file 1. **Concentration and time of Free Fatty Acid induced ER Stress in L02 hepatocyte. (A): Effects of FFA at different concentrations on the growth of L02 cells. (B): Lipid content was measured by ORO-based colorimetric assay. (C): Lipid droplets were observed by Oil Red O staining (20 × 10magnification). (D): The level of triglyceride in LO2 cells treated by 1.0 mM FFA for different time. (E, F, G, and H): The expression of GRP78, CHOP, and CASPASE12 protein and mRNA in L02 cells treated by 1 mM FFA for different time (0, 6, 12, 24, 48 h). Results are expressed as means ± S.D. ^*^p < 0.05, ^**^p < 0.01 compared with the control group.

**Additional file 2.** Blot images.

## Data Availability

The datasets used and/or analyzed during the current study available from the corresponding author on reasonable request.

## Abbreviations

ATF4: Activating Transcription Factor 4
ATF6: Activating Transcription Factor 6
CANX: Canlexin
CASPASE12/3: Cysteinyl aspartate specific proteinase 12/3
CHOP: C/EBP homologous protein
ERS: Endoplasmic reticulum stress
FFA: Free fatty acid
FOXO1: Forkhead box O1
GRP78: Glucose-regulated protein 78
HQT: Hugan Qingzhi tablet
IP3R: 1,4,5-trisphosphate receptor
IRE1: Inositol-requiring Transmembrane Kinase Type-I
LHD: Lactate dehydrogenase
NAFLD: Non-alcoholic fatty liver disease
NAFL: Nonalcoholic simple fatty liver
NASH: Nonalcoholic steatohepatitis
PERK: Protein Kinase-like ER Kinase
PKC-δ: Protein kinase C-δ
ROS: Reactive oxygen species
SERCA: Sarco endoplasmic reticulum calcium adenosine triphosphatase
TG: Triglyceride
UPR: Unfolded protein reaction
XBP-1: X box-binding protein-1.

## References

[1] Brent AN (2017). Non-alcoholic fatty liver disease. BMC Med.

[2] Day CP, James OFW (1998). Steatohepatitis: a tale of two “hits”?. Gastroenterology.

[3] Gentile CL, Pagliassotti MJ (2008). The role of fatty acids in the development and progression of nonalcoholic fatty liver disease. J Nutr Biochem.

[4] Hetz C, Chevet E, Oakes SA (2015). Proteostasis control by the unfolded protein response. Nat Cell Biol.

[5] Zhang X (2014). Role of endoplasmic reticulum stress in the pathogenesis of nonalcoholic fatty liver disease. World J Gastroenterol.

[6] Ji C (2008). Dissection of endoplasmic reticulum stress signaling in alcoholic and non-alcoholic liver injury. J Gastroenterol Hepatol.

[7] Afrin R, Arumugam S, Wahed MII, Pitchaimani V, Karuppagounder V, Sreedhar R, Harima M, Suzuki H, Miyashita S, Nakamura T (2016). Attenuation of endoplasmic reticulum stress-mediated liver damage by mulberry leaf diet in Streptozotocin-induced diabetic rats. Am J Chin Med.

[8] Newton AC (2010). Protein kinase C: poised to signal. Am J Physiol Endoc Metab.

[9] Bezy O, Tran TT, Pihlajamäki J, Suzuki R, Emanuelli B, Winnay J, Mori MA, Haas J, Biddinger SB, Leitges M (2011). PKCδ regulates hepatic insulin sensitivity and hepatosteatosis in mice and humans. J Clin Invest.

[10] Greene MW, Burrington CM, Luo Y, Ruhoff MS, Lynch DT, Chaithongdi N (2014). PKCδ is activated in the liver of obese Zucker rats and mediates diet-induced whole body insulin resistance and hepatocyte cellular insulin resistance. J Nutr Biochem.

[11] Greene MW, Burrington CM, Lynch DT, Davenport SK, Johnson AK, Horsman MJ, Chowdhry S, Zhang J, Sparks JD, Tirrell PC (2014). Lipid metabolism, oxidative stress and cell death are regulated by PKC delta in a dietary model of nonalcoholic steatohepatitis. PLoS One.

[12] Greene MW, Burrington CM, Ruhoff MS, Johnson AK, Chongkrairatanakul T, Kangwanpornsiri A (2010). PKCδ is activated in a dietary model of Steatohepatitis and regulates endoplasmic reticulum stress and cell death. J Biol Chem.

[13] Hamza AA, Heeba GH, Elwy HM, Murali C, El-Awady R, Amin A (2018). Molecular characterization of the grape seeds extract's effect against chemically induced liver cancer: in vivo and in vitro analyses. Sci Rep.

[14] Al-Dabbagh B, Elhaty IA, Al HA, Al SR, El-Awady R, Ashraf SS, Amin A (2018). Antioxidant and anticancer activities of Trigonella foenum-graecum, Cassia acutifolia and Rhazya stricta. BMC Complement Altern Med.

[15] Al-Dabbagh B, Elhaty IA, Elhaw M, Murali C, Al MA AB, Amin A (2019). Antioxidant and anticancer activities of chamomile (*Matricaria recutita* L.). BMC Res Notes.

[16] El-Dakhly SM, Salama A, Hassanin S, Yassen NN, Hamza AA, Amin A (2020). Aescin and diosmin each alone or in low dose- combination ameliorate liver damage induced by carbon tetrachloride in rats. BMC Res Notes.

[17] Yin J, Luo Y, Deng H, Qin S, Tang W, Zeng L, Zhou B (2014). Hugan Qingzhi medication ameliorates hepatic steatosis by activating AMPK and PPARα pathways in L02 cells and HepG2 cells. J Ethnopharmacol.

[18] Tang W, Yao X, Xia F, Yang M, Chen Z, Zhou B, Liu Q (2018). Modulation of the gut microbiota in rats by Hugan Qingzhi tablets during the treatment of high-fat-diet-induced nonalcoholic fatty liver disease. Oxidative Med Cell Longev.

[19] Xiao C, He F, Yang M, Xia F, Tang W, Zhou B, Zhou H (2018). Ultra-high performance liquid chromatography/triple quadrupole mass spectrometry study of the stabilities and transformations of four alisols in Alismatis Rhizoma and proprietary traditional Chinese medicine prescriptions. Pharmacogn Mag.

[20] Jinjin Y, Waijiao T, Lu Z, Benjie Z (2014). Establishment of a L-02 cell model of hepatic steatosis (in chinese). J Southern Med Univ.

[21] Xiaorui Y, Fan X, Waijiao T, Benjie Z (2017). Effect of Hugan Qingzhi tablets on AMPK pathway activation and NF-κB-p65 protein expression in the liver of rats with nonalcoholic fatty liver disease (in chinese). In..

[22] Tang W, Zhou B, Zhou H (2013). Study on pharmacodynamics of Hugan Qingzhi tablet on non-alcoholic fatty liver in rats (in chinese). Pharmacol Clin Chin Mater Med.

[23] Tang W, Zeng L, Yin J, Yao Y, Feng L, Yao X, Sun X, Zhou B (2015). Hugan Qingzhi exerts anti-inflammatory effects in a rat model of nonalcoholic fatty liver disease. Evid-Based Complement Alternat Med.

[24] Waijiao T, Yufa Y, JInjin Y, Lu Z, Benjie Z (2014). Effects of Hugan Qingzhi tablet on liver protein synthesis and bile metabolism in rats with non-alcoholic fatty liver (in chinese). J Chin Med Mater.

[25] Xia F, Yao X, Tang W, Xiao C, Yang M, Zhou B (2017). Isobaric tags for relative and absolute quantitation (iTRAQ)-based proteomic analysis of Hugan Qingzhi and its protective properties against free fatty acid-induced L02 hepatocyte injury. Front Pharmacol.

[26] Yao X, Xia F, Tang W, Xiao C, Yang M, Zhou B (2018). Isobaric tags for relative and absolute quantitation (iTRAQ)-based proteomics for the investigation of the effect of Hugan Qingzhi on non-alcoholic fatty liver disease in rats. J Ethnopharmacol.

[27] Perumpail BJ, Khan MA, Yoo ER, Cholankeril G, Kim D, Ahmed A (2017). Clinical epidemiology and disease burden of nonalcoholic fatty liver disease. World J Gastroenterol.

[28] Maher JJ. Pathogenesis of NAFLD and NASH. In: Chalasani N, Szabo G, editors, Alcoholic and non-alcoholic fatty liver disease. Switzerland; 2016. p. 71–101. 10.1007/978-3-319-20538-0_4.

[29] Ge J, Wang D, He R, Zhu H, Wang Y, He S (2010). Medicinal herb research: serum pharmacological method and plasma pharmacological method. Biol Pharm Bull.

[30] Vidyashankar S, Sandeep Varma R, Patki PS (2013). Quercetin ameliorate insulin resistance and up-regulates cellular antioxidants during oleic acid induced hepatic steatosis in HepG2 cells. Toxicol in Vitro.

[31] Soardo G, Donnini D, Domenis L, Catena C, De Silvestri D, Cappello D, Dibenedetto A, Carnelutti A, Bonasia V, Pagano C (2011). Oxidative stress is activated by free fatty acids in cultured human hepatocytes. Metab Syndr Relat Disord.

[32] Gentile CL, Frye M, Pagliassotti MJ. Endoplasmic reticulum stress and the unfolded protein response in nonalcoholic fatty liver disease. Antioxid Redox Sign. 2011;15(2):505–21. 10.1089/ars.2010.3790 PMC3118611 21128705

[33] Malhi H, Kaufman RJ (2011). Endoplasmic reticulum stress in liver disease. J Hepatol.

[34] Bobrovnikova-Marjon E, Hatzivassiliou G, Grigoriadou C, Romero M, Cavener DR, Thompson CB, Diehl JA (2008). PERK-dependent regulation of lipogenesis during mouse mammary gland development and adipocyte differentiation. Proc Natl Acad Sci U S A.

[35] Kaplowitz N, Than TA, Shinohara M, Ji C (2007). Endoplasmic reticulum stress and liver injury. Semin Liver Dis.

[36] Yeh K, Lai C, Lin C, Hsu C, Lo C, Her GM (2017). ATF4 overexpression induces early onset of hyperlipidaemia and hepatic steatosis and enhances adipogenesis in zebrafsh. Sci Rep.

[37] Xiao G, Zhang T, Yu S, Lee S, Calabuig-Navarro V, Yamauchi J, Ringquist S, Dong HH (2013). ATF4 protein deficiency protects against high fructose-induced hypertriglyceridemia in mice. J Biol Chem.

[38] Wang S, Kaufman RJ (2014). How does protein misfolding in the endoplasmic reticulum affect lipid metabolism in the liver?. Curr Opin Lipidol.

[39] So J, Hur KY, Tarrio M, Ruda V, Frank-Kamenetsky M, Fitzgerald K, Koteliansky V, Lichtman AH, Iwawaki T, Glimcher LH (2012). Silencing of lipid metabolism genes through IRE1α-mediated mRNA decay lowers plasma lipids in mice. Cell Metab.

[40] Sha H, He Y, Chen H, Wang C, Zenno A, Shi H, Yang X, Zhang X, Qi L (2009). The IRE1α-XBP1 pathway of the unfolded protein response is required for Adipogenesis. Cell Metab.

[41] Maruyama R, Kamoshida Y, Shimizu M, Inoue J, Sato R (2013). ATF6α stimulates Cholesterogenic gene expression and de novo cholesterol synthesis. Biosci Biotechol Biochem.

[42] Mollica MP, Lionetti L, Putti R, Cavaliere G, Gaita M, Barletta A (2011). From chronic overfeeding to hepatic injury_ role of endoplasmic reticulum stress and inflammation. Nutr Metab Cardiovasc Dis.

[43] Tamaki N, Hatano E, Taura K, Tada M, Kodama Y, Nitta T, Iwaisako K, Seo S, Nakajima A, Ikai I (2008). CHOP deficiency attenuates cholestasis-induced liver fibrosis by reduction of hepatocyte injury. Am J Physiol Gastrointest Liver Physiol.

[44] Ji C, Mehrian Shai R, Chan C, Hsu YH, Kaplowitz N (2005). Role of CHOP in hepatic apoptosis in the murine model of Intragastric ethanol feeding. Alcohol Clin Exp Res.

[45] Yoneda T, Imaizumi K, Oono K, Yui D, Gomi F, Katayama T, Tohyama M (2001). Activation of caspase-12, an endoplastic reticulum (ER) resident caspase, through tumor necrosis factor receptor-associated factor 2-dependent mechanism in response to the ER stress. J Biol Chem.

[46] Hetz C (2006). Proapoptotic BAX and BAK modulate the unfolded protein response by a direct interaction with IRE1. Science.

[47] Arruda AP, Pers BM, Parlakgül G, Güney E, Inouye K, Hotamisligil GS (2014). Chronic enrichment of hepatic endoplasmic reticulum-mitochondria contact leads to mitochondrial dysfunction in obesity. Nat Med.

[48] Rieusset J (2017). Endoplasmic reticulum-mitochondria calcium signalling in hepatic metabolic diseases. Mol Cell Res.

[49] Al-Hrout A, Chaiboonchoe A, Khraiwesh B, Murali C, Baig B, El-Awady R, Tarazi H, Alzahmi A, Nelson DR, Greish YE (2018). Safranal induces DNA double-strand breakage and ER-stress-mediated cell death in hepatocellular carcinoma cells. Sci Rep.

[50] Schmitz-Peiffer C, Biden TJ (2008). Protein kinase C function in muscle, liver, and -cells and its therapeutic implications for type 2 Diabetes. Diabetes.

[51] Lee SJ, Kang JH, Choi SY, Suk KT, Kim DJ, Kwon O (2013). PKCδ as a regulator for TGFb1-induced a-SMA production in a murine nonalcoholic steatohepatitis model. PLoS One.

[52] Klymenko K, Novokhatska T, Kizub I, Parshikov A, Dosenko V, Soloviev A (2014). PKC-delta isozyme gene silencing restores vascular function in diabetic rat. J Basic Clin Physiol Pharmacol.

[53] Lai S, Li Y, Kuang Y, Cui H, Yang Y, Sun W, Liu K, Chen D, Yan Q, Wen L (2017). PKCδ silencing alleviates saturated fatty acid induced ER stress by enhancing SERCA activity. Biosci Rep.

[54] Luciani DS, Gwiazda KS, Yang TLB, Kalynyak TB, Bychkivska Y, Frey MHZ, Jeffrey KD, Sampaio AV, Underhill TM, Johnson JD (2009). Roles of IP3R and RyR Ca2+ channels in endoplasmic reticulum stress and cell death. Diabetes.

[55] Wong VK, Li T, Law BY, Ma ED, Yip NC, Michelangeli F, Law CK, Zhang MM, Lam KY, Chan PL (2013). Saikosaponin-d, a novel SERCA inhibitor, induces autophagic cell death in apoptosis-defective cells. Cell Death Dis.

[56] De Ford C, Heidersdorf B, Haun F, Murillo R, Friedrich T, Borner C, Merfort I (2016). The clerodane diterpene casearin J induces apoptosis of T-ALL cells through SERCA inhibition, oxidative stress, and interference with Notch1 signaling. Cell Death Dis.

[57] Liang C, Han S, Li G, Tabas I, Tall AR (2012). Impaired MEK signaling and SERCA expression promote ER stress and apoptosis in insulin-resistant macrophages and are reversed by Exenatide treatment. Diabetes.

[58] Zhang J, Li Y, Jiang S, Yu H, An W (2014). Enhanced endoplasmic reticulum SERCA activity by overexpression of hepatic stimulator substance gene prevents hepatic cells from ER stress-induced apoptosis. Am J Physiol Cell Physiol.

[59] Cunha DA, Hekerman P, Ladriere L, Bazarra-Castro A, Ortis F, Wakeham MC, Moore F, Rasschaert J, Cardozo AK, Bellomo E (2008). Initiation and execution of lipotoxic ER stress in pancreatic -cells. J Cell Sci.

[60] Tong X, Kono T, Evans-Molina C (2015). Nitric oxide stress and activation of AMP-activated protein kinase impair β-cell sarcoendoplasmic reticulum calcium ATPase 2b activity and protein stability. Cell Death Dis.

