# Supplementary figures and images for: Hugan Qingzhi medication ameliorates free fatty acid-induced L02 hepatocyte endoplasmic reticulum stress by regulating the activation of PKC-δ
Source: BMC Complement Med Ther. 2020 Dec 11;20:377. doi: 10.1186/s12906-020-03164-3 (PMC7730760; doi:10.1186/s12906-020-03164-3)

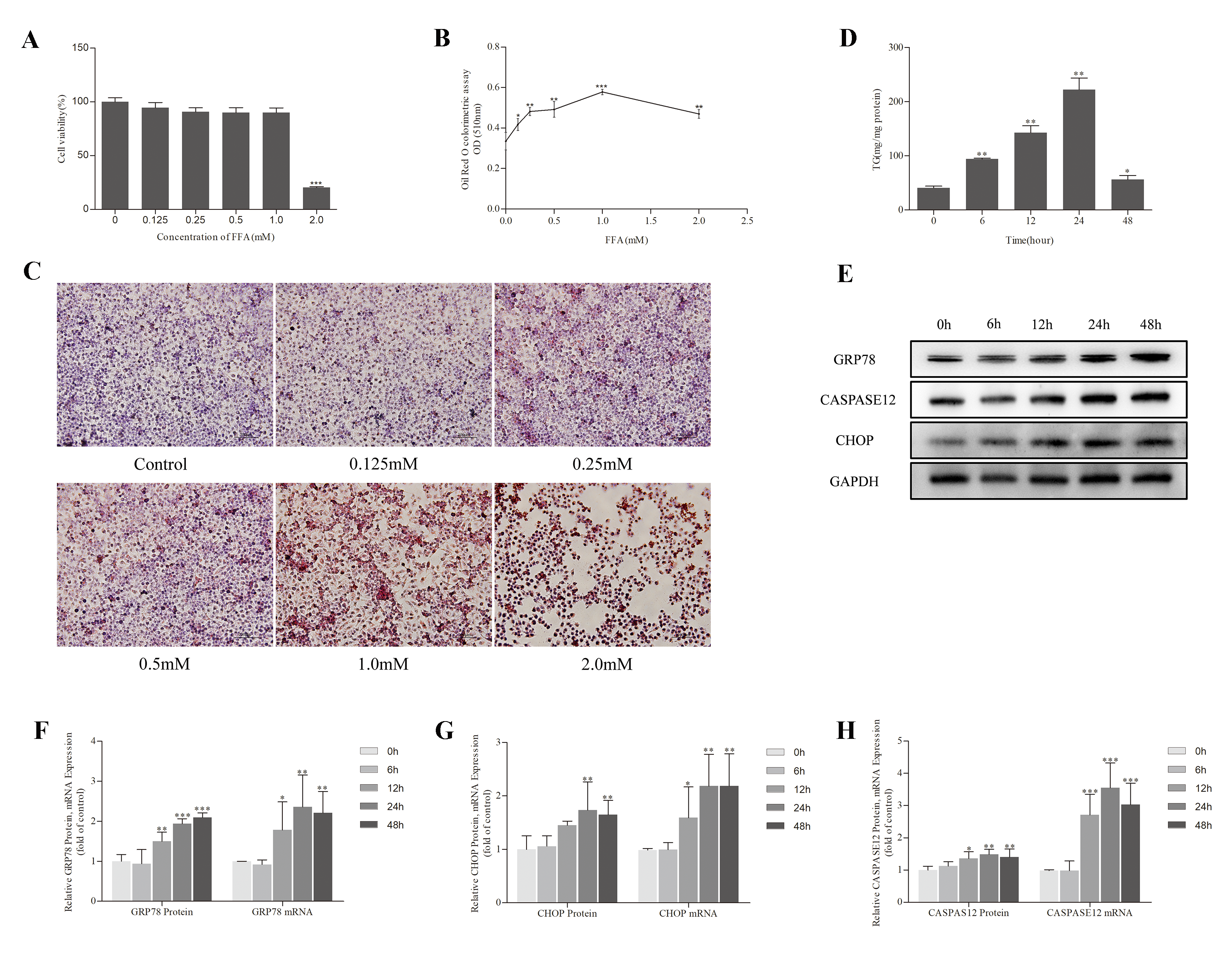

Supplement: Supplementary file 1 — Additional file 1. Concentration and time of Free Fatty Acid induced ER Stress in L02 hepatocyte. (A): Effects of FFA at different concentrations on the growth of L02 cells. (B): Lipid content was measured by ORO-based colorimetric assay. (C): Lipid droplets were observed by Oil Red O staining (20 × 10magnification). (D): The level of triglyceride in LO2 cells treated by 1.0 mM FFA for different time. (E, F, G, and H): The expression of GRP78, CHOP, and CASPASE12 protein and mRNA in L02 cells treated by 1 mM FFA for different time (0, 6, 12, 24, 48 h). Results are expressed as means ± S.D. *p < 0.05, **p < 0.01 compared with the control group. [file 12906_2020_3164_MOESM1_ESM.tif]
