# Supplementary material for: Hugan Qingzhi medication ameliorates free fatty acid-induced L02 hepatocyte endoplasmic reticulum stress by regulating the activation of PKC-δ
Source: BMC Complement Med Ther. 2020 Dec 11;20:377. doi: 10.1186/s12906-020-03164-3 (PMC7730760; doi:10.1186/s12906-020-03164-3)
Supplement: Supplementary file 2 — Additional file 2. Blot images. [file 12906_2020_3164_MOESM2_ESM.pdf]

Additional file.1 Blot images

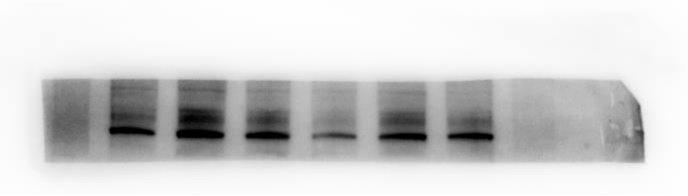

Figure.2B PERK

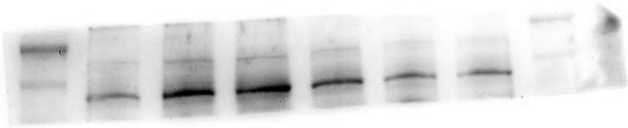

Figure.2B p-PERK

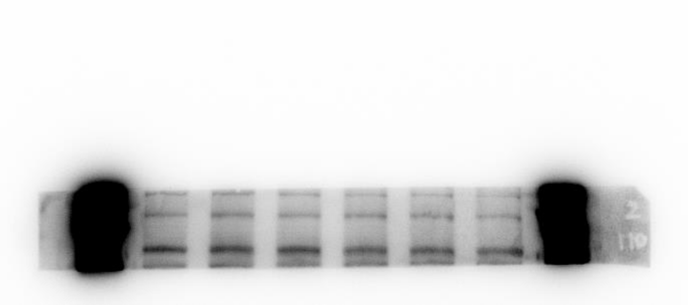

Figure.2B IRE-1α

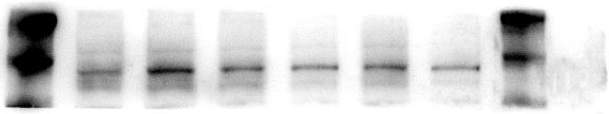

Figure.2B p-IRE-1α

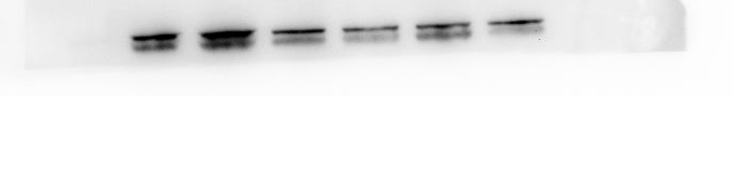

Figure.2B GRP78

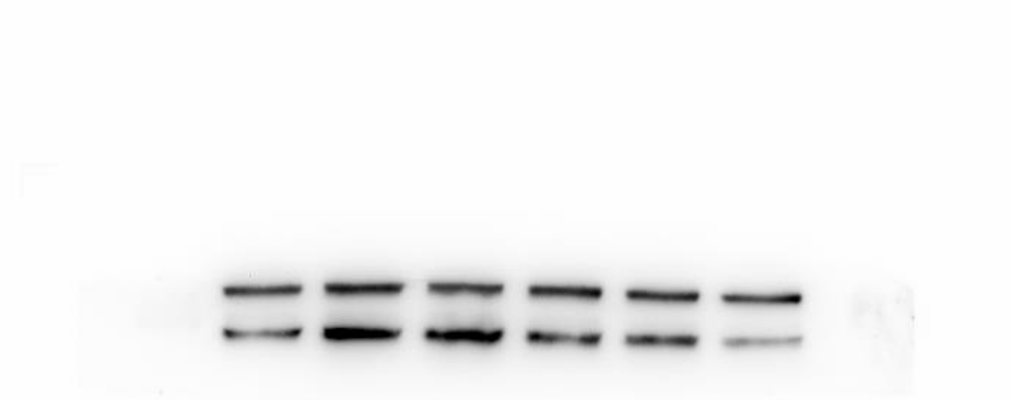

Figure.2B ATF6

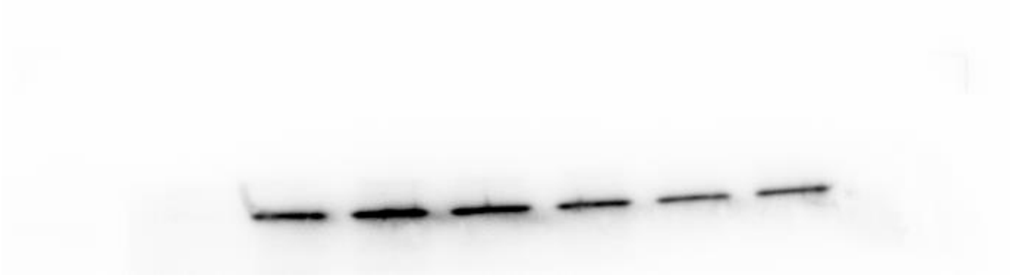

Figure.2B ATF4

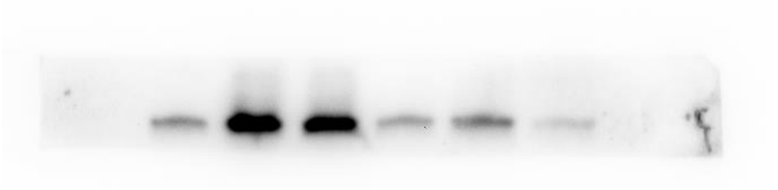

Figure.2B CASPASE12

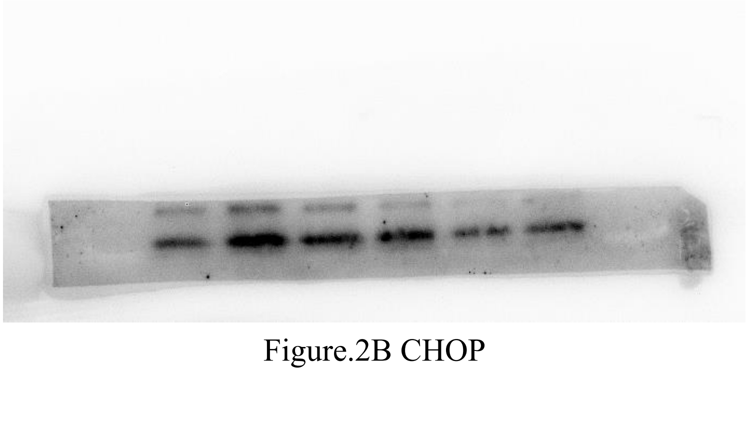

Figure.2B CHOP

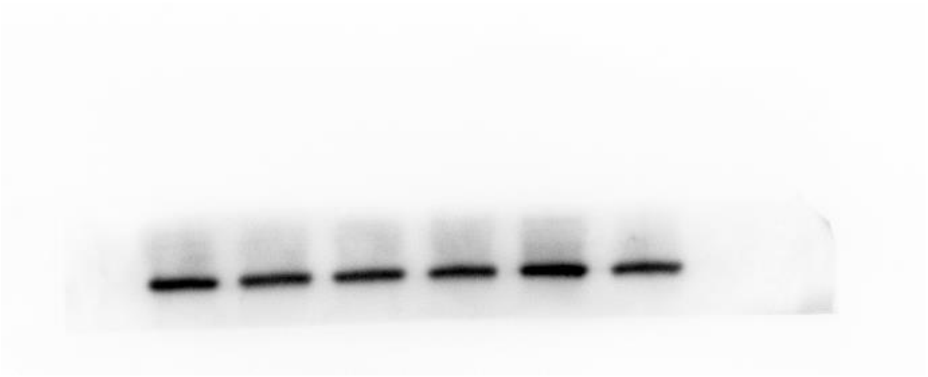

Figure.2B GAPDH

Fig.2 HQT-medicated serum protects against FFA-induced L02 hepatocyte from ERS. (**B**): The expression levels of PERK, p-PERK, IRE-1α, p-IRE-1α, GRP78, ATF6, ATF4, CASPASE12, CHOP protein

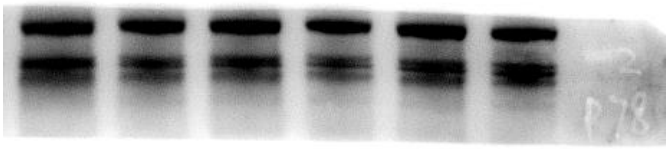

Figure.3A PKC-δ

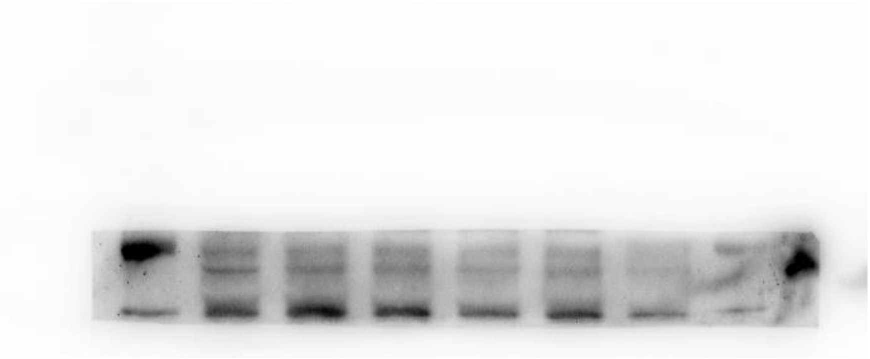

Figure.3 Ap-PKC-δ

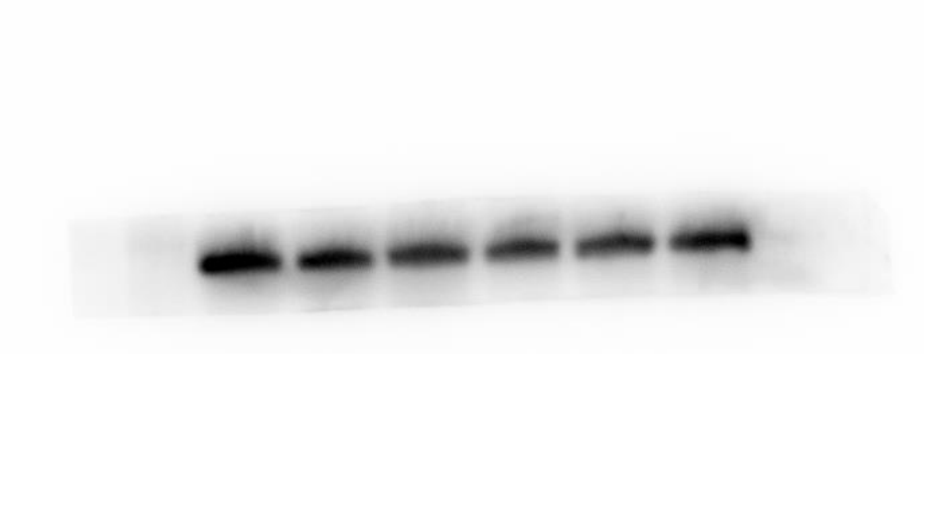

Figure.3A GAPDH

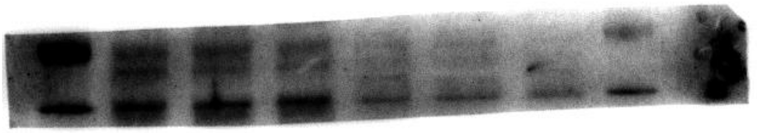

Figure.3C PKC-δ

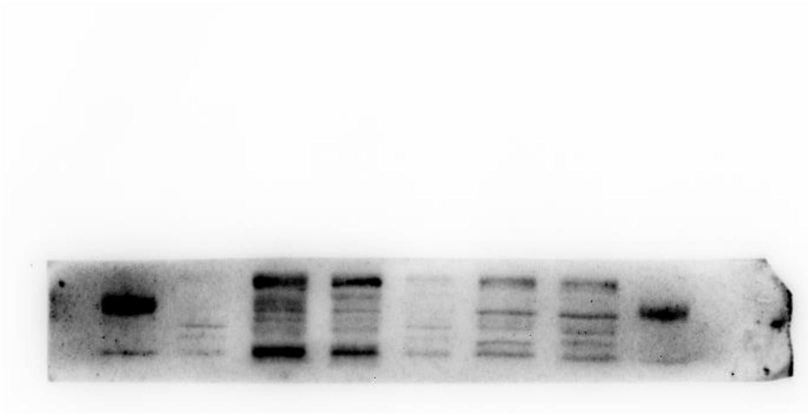

Figure.3C p-PKC-δ

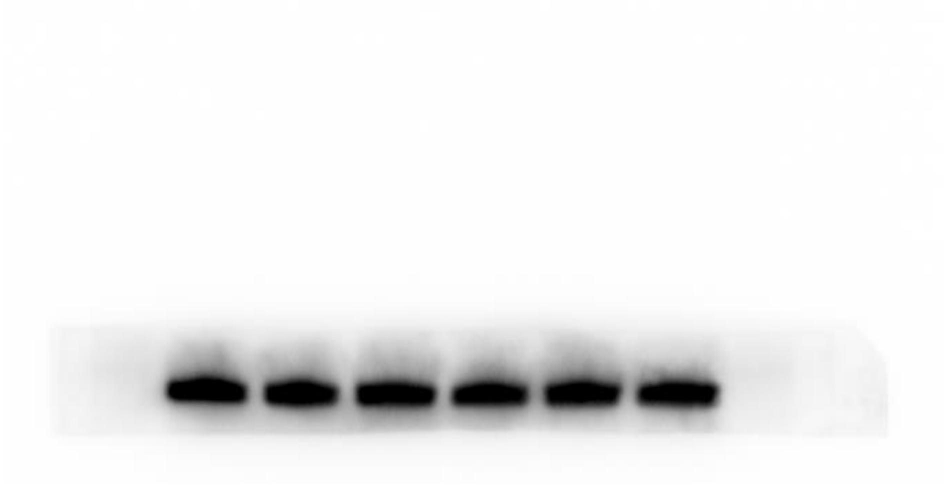

Figure.3C GDPDH

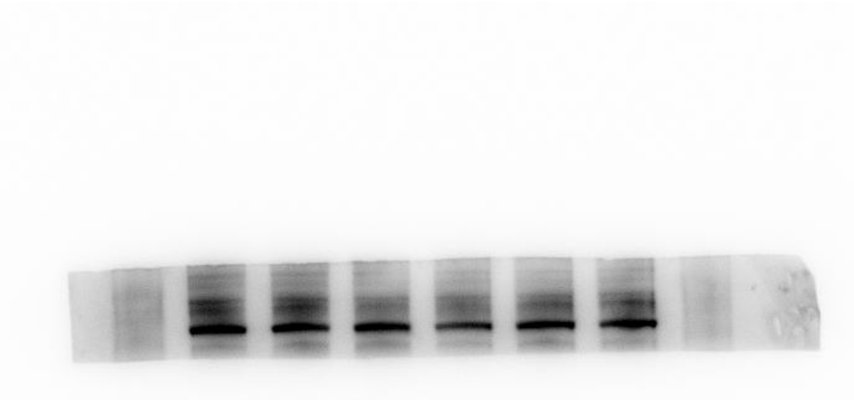

Figure.3E PERK

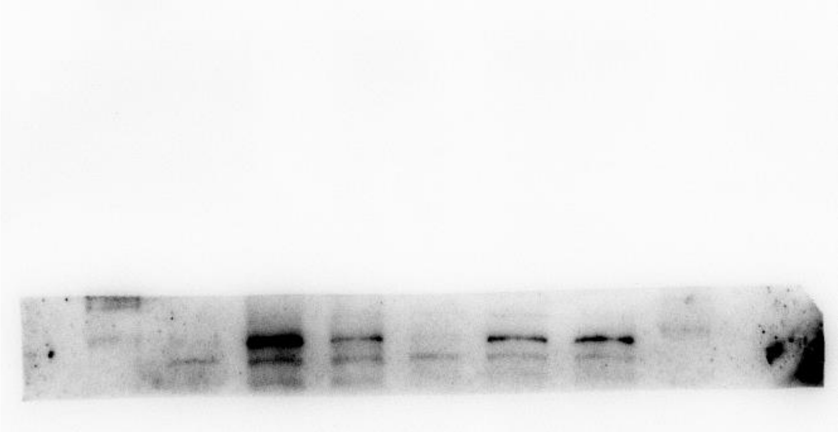

Figure.3E p-PERK

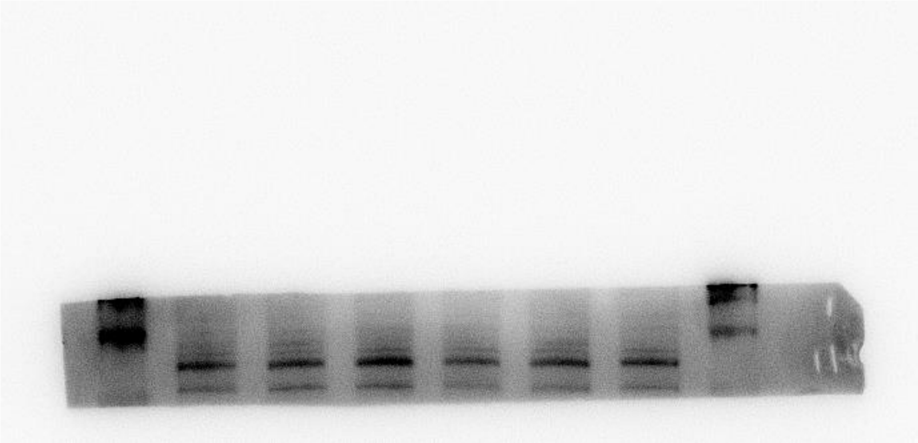

Figure.3E IRE-1α

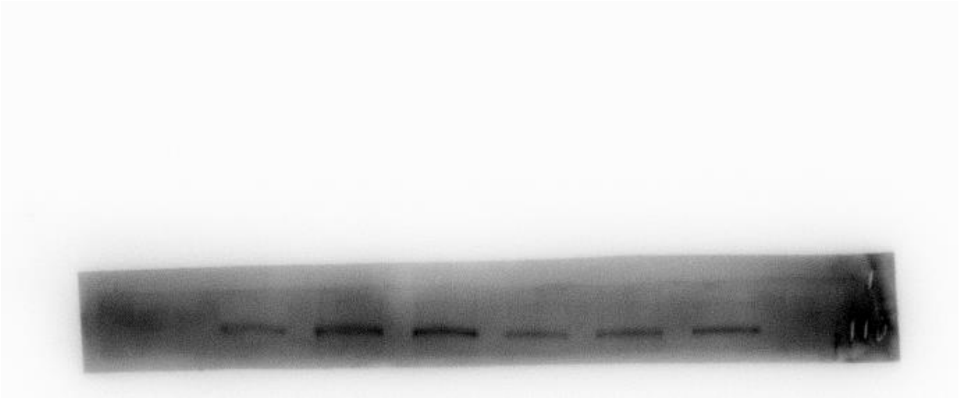

Figure.3E p-IRE-1α

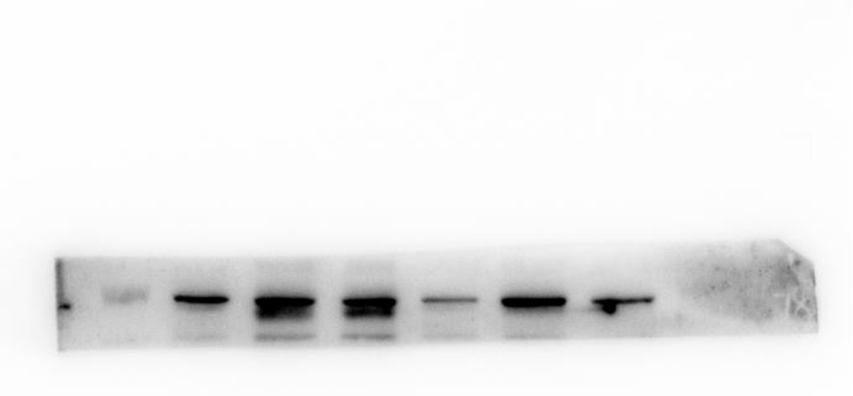

Figure.3E GRP78

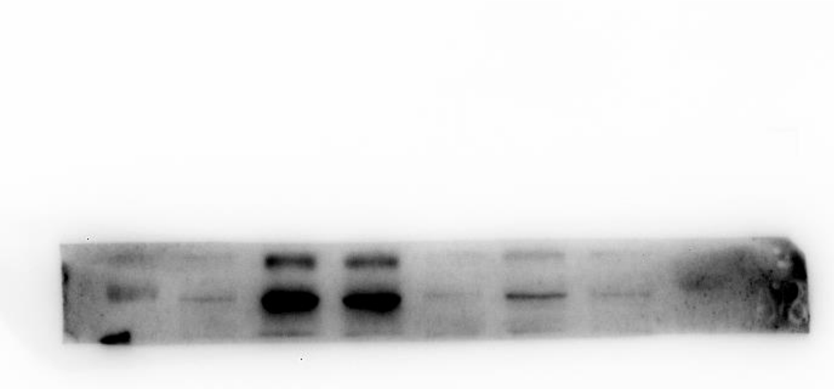

Figure.3E ATF6

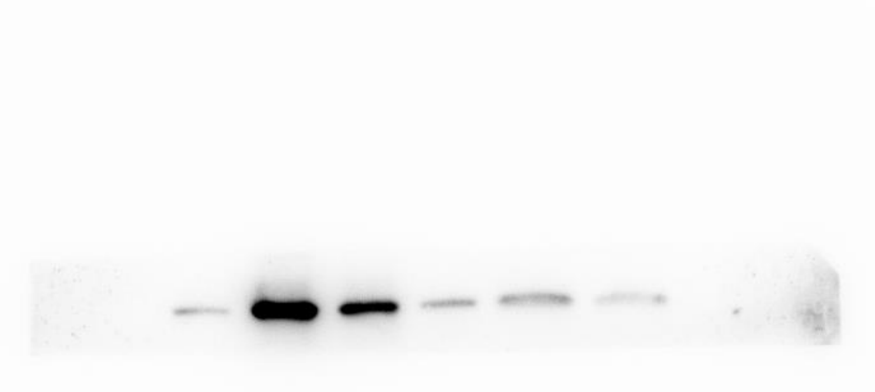

Figure.3E ATF4

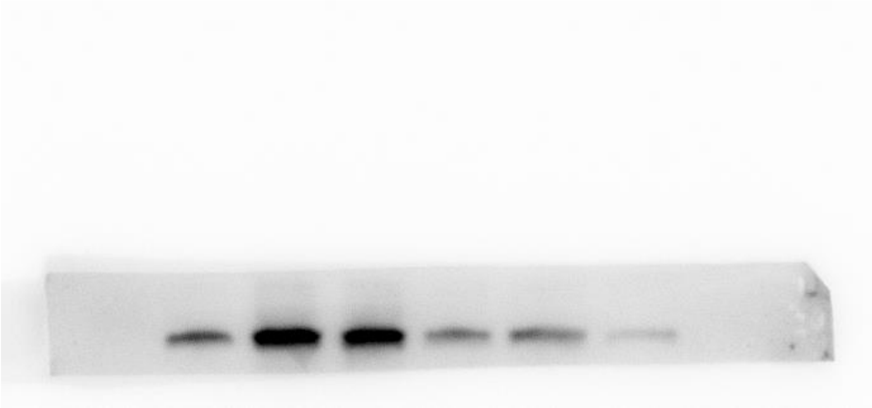

Figure.3E CASPASE12

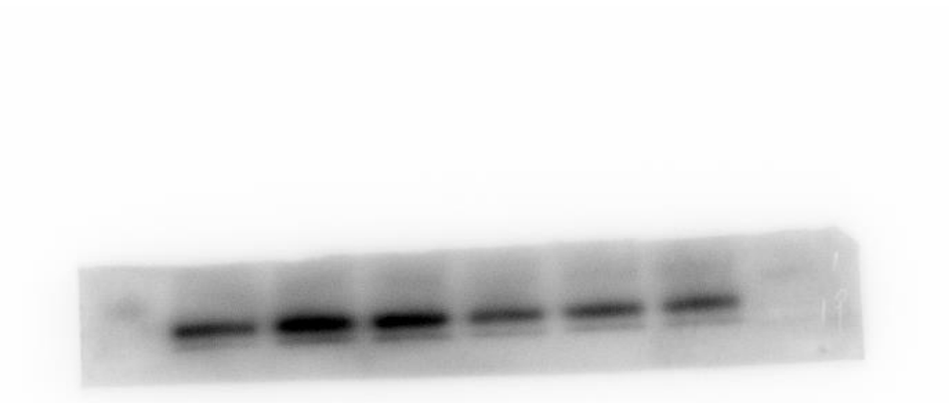

Figure.3E CHOP

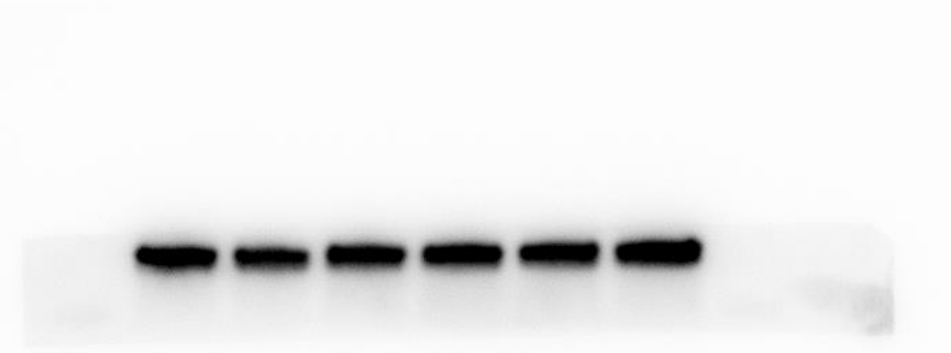

Figure.3E GAPDH

Fig.3 PKC-δ silencing ameliorates FFA-induced L02 hepatocyte ERS. (**E**): Effects of HQT-medicated serum on the expression levels of p-PERK, p-IRE-1α, GRP78, ATF6, ATF4, CASPASE12, CHOP protein in FFA-stimulated L02 hepatocytes with or without transfection of PKC-δ siRNA.

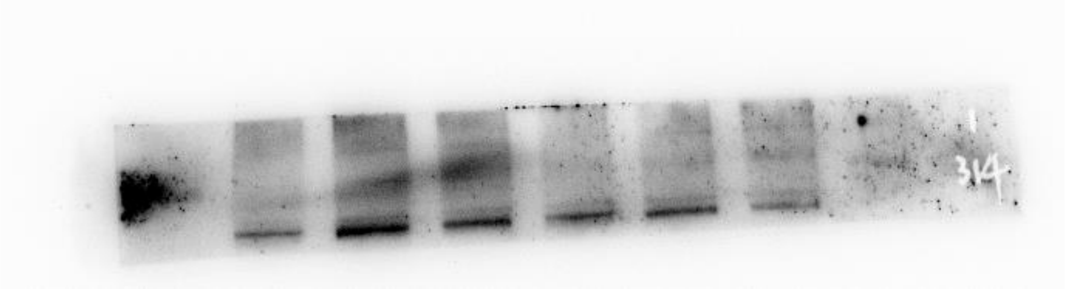

Figure.4C IP3R

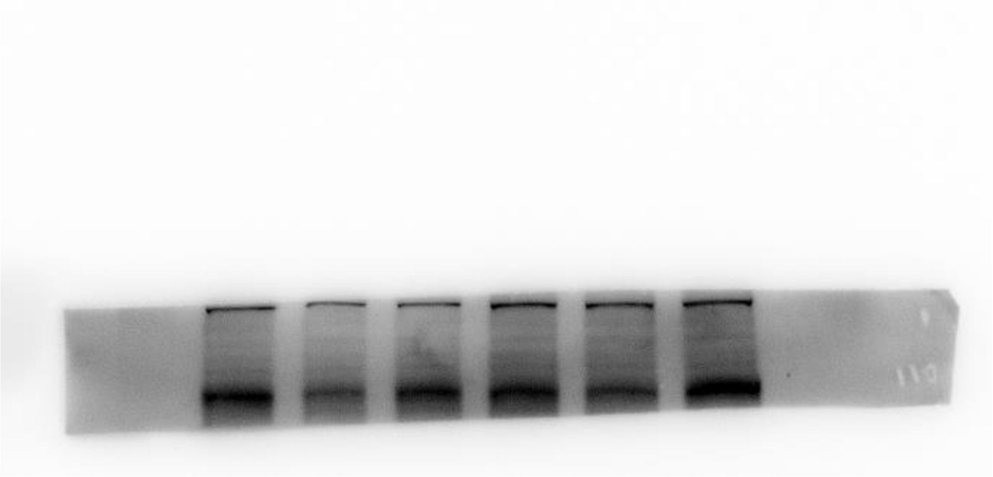

Figure.4C SERCA2

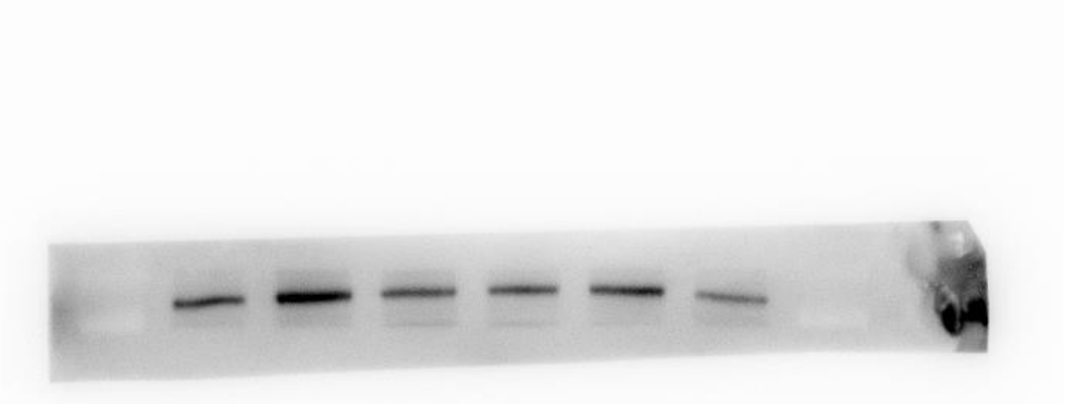

Figure.4C CANX

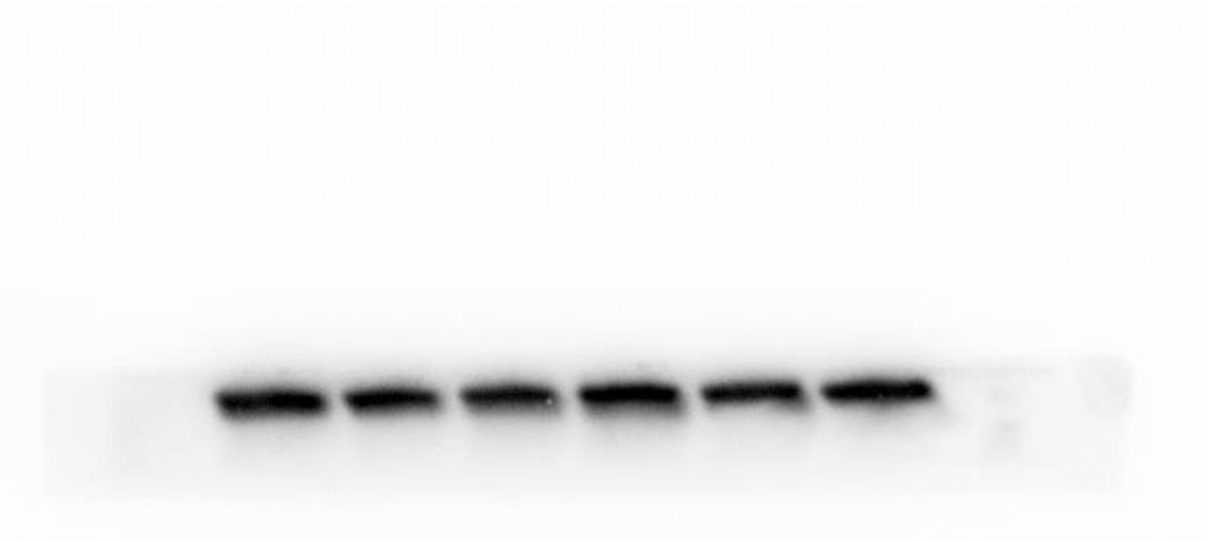

Figure.4C GAPDH

Fig.4 PKC-δ silencing recovers Calcium homeostasis in ER. (**C**): The expression levels of SERCA2, CANX, and IP3R protein in L02 cells with or without transfection of PKC-δ siRNA.

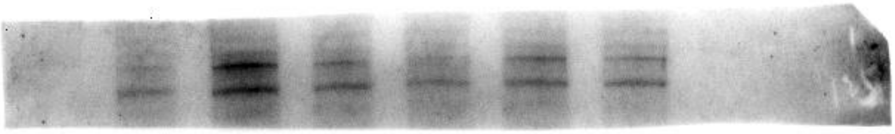

Figure.5D SREBP1C

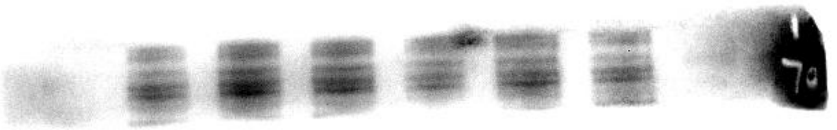

Figure.5D FOXO1

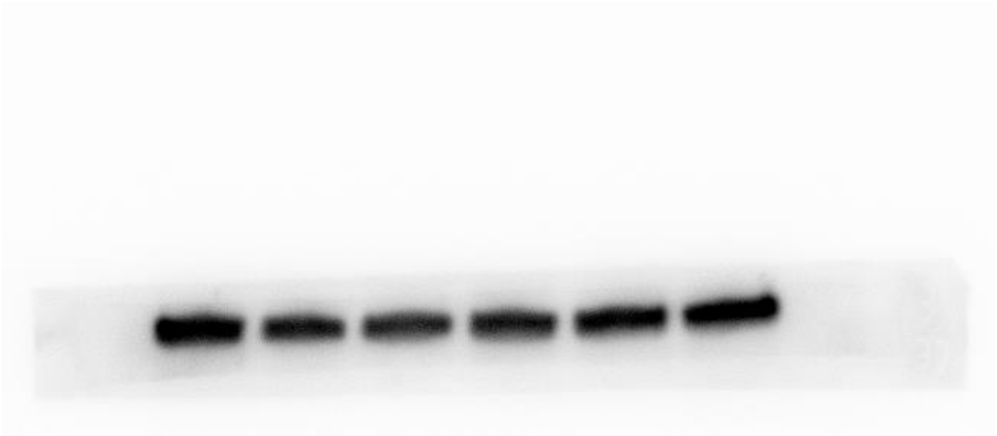

Figure.5D GAPDH

Fig.5 PKC-δ silencing inhibits FFA-induced L02 hepatocyte steatosis. (**D**): The expression levels of SREBP-1C and FOXO1 protein in L02 cells with or without transfection of PKC-δ siRNA.

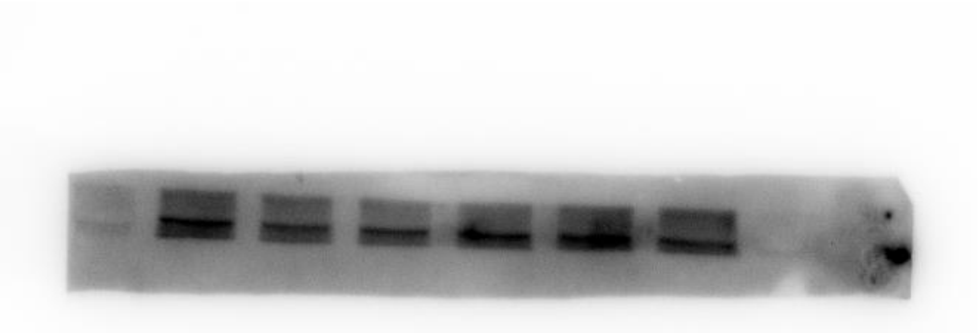

Figure.6F PI3K-p85

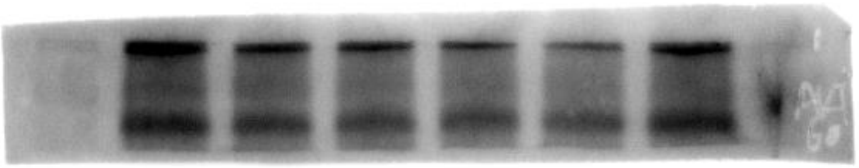

Figure.6F AKT

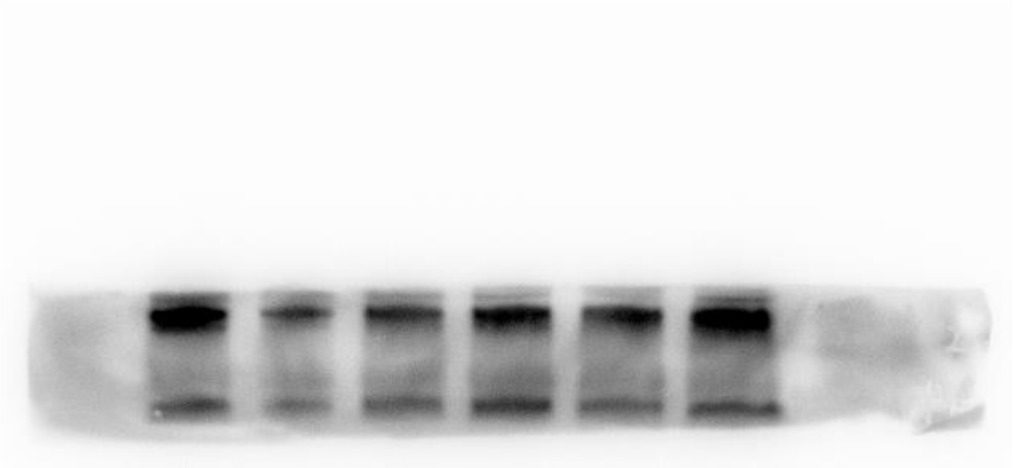

Figure.6F p-AKT

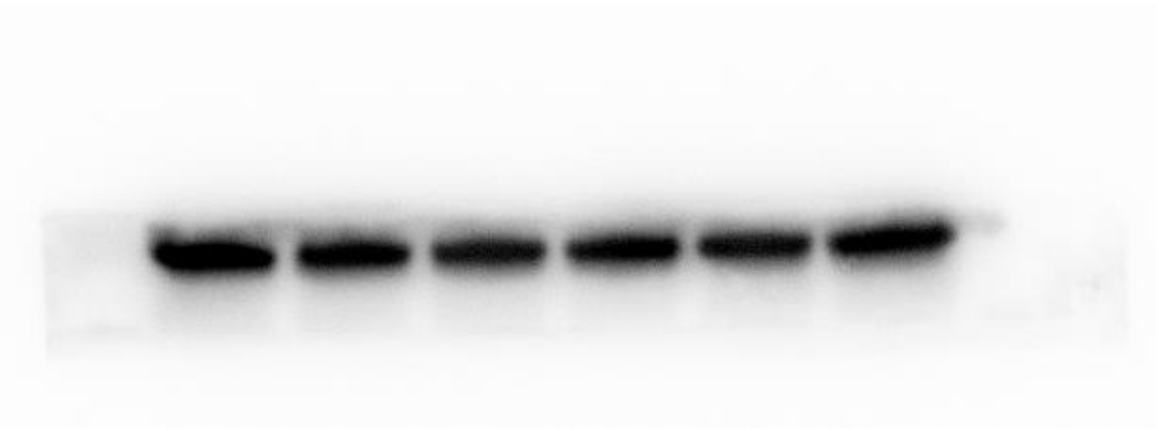

Figure.6F GAPDH

Fig.6 PKC-δ silencing improves insulin resistance in FFA-induced L02 hepatocyte. (**F**) The expression levels of PI3K-p85, AKT, and p-AKT protein in L02 cells with or without transfection of PKC-δ siRNA.

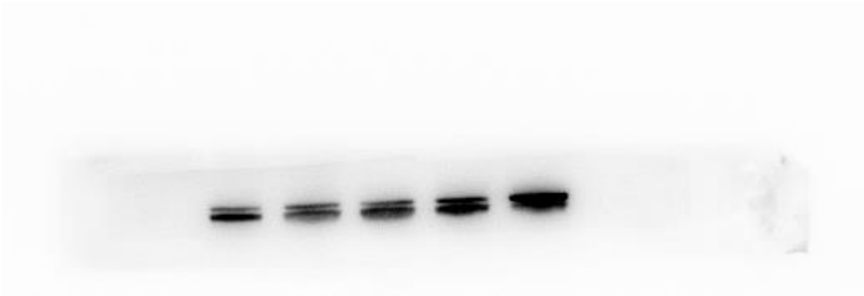

Figure.7E GRP78

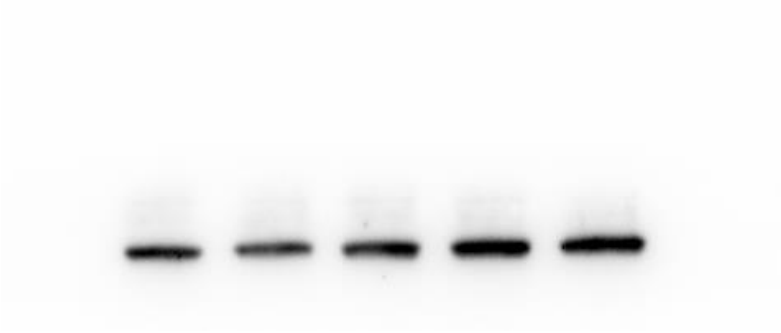

Figure.7E CASPASE12

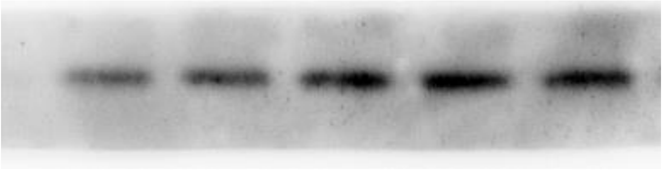

Figure.7E CHOP

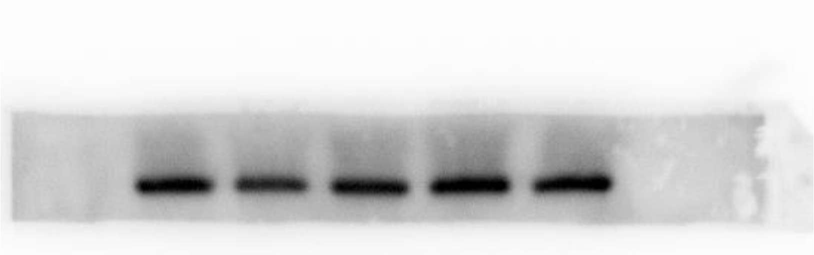

Figure.7E GAPDH

Figure.7 Concentration and time of Free Fatty Acid induced ER Stress in L02 hepatocyte. (**E**): The expression of GRP78, CHOP, and CASPASE12 protein in L02 cells treated by 1mM FFA for different time (0, 6, 12, 24, 48h).
